# Comparative effectiveness of school-based exercise interventions on physical fitness in children and adolescents: a systematic review and network meta-analysis

**DOI:** 10.3389/fpubh.2023.1194779

**Published:** 2023-06-05

**Authors:** Jin Wu, Yuhang Yang, Huasen Yu, Liqiang Li, Yanying Chen, Youping Sun

**Affiliations:** ^1^College of Physical Education and Health, East China Normal University, Shanghai, China; ^2^Business School, NingboTech University, Ningbo, China

**Keywords:** adolescents, school-based exercise, physical fitness, children, network meta-analysis

## Abstract

**Background:**

Schools provide a favorable setting for health education, however, the most effective school-based exercise mode for improving physical fitness remains unclear. This network meta-analysis was designed to assess and rank the comparative efficacy of six exercise modalities on physical fitness indicators in a school-based setting.

**Methods:**

An online search of the Web of Science, PubMed, SPORTDiscus, and Scopus databases was conducted. Randomized and quasi-randomized controlled trials were considered. Outcomes included measures of anthropometry and body composition, muscular fitness, and cardiorespiratory fitness. Data were pooled with a random effects model using the frequentist framework.

**Results:**

A total of 66 studies with 8,578 participants (48% girls) were included. High-intensity interval training was the most effective intervention reducing body mass index (mean difference (MD) = −0.60 kg·m^−2^, 95% confidence interval (95%CI) = −1.04 to −0.15, *p* = 0.009), elevating VO_2max_ (MD = 3.59 mL·kg^−1^·min^−1^, 95% CI = 2.45 to 4.74, *p* < 0.001), and 20-meter sprint performance (MD = −0.35 s, 95% CI = −0.55 to −0.14, *p* = 0.001). Aerobic training had the highest probability of reducing waist circumference (standardized mean difference (SMD) = −0.60, 95% CI = −0.88 to −0.32, *p* < 0.001). Active video games emerged as a promising modality for improving countermovement jump (MD = 2.43 cm, 95% CI = 0.06 to 4.80, *p* = 0.041) and shuttle running performance (SMD = 0.86, 95% CI = 0.29 to 1.43, *p* = 0.003). Strength training was the best exercise mode for improving standing long jump performance (SMD = 1.03, 95% CI = 0.07 to 1.98, *p* = 0.035) while combined training was rated the first for decreasing body fat percent (MD = −2.56%, 95% CI = −4.73 to −0.40, *p* = 0.022) and increasing push-up repetitions (SMD = 3.59, 95% CI = 0.81 to 6.37, *p* = 0.012).

**Conclusion:**

School-based exercise interventions have multiple effects on physical fitness. The findings of this study will help to inform physical education teachers and coaches how best to deliver exercise programs in a school setting. Since the study was limited by the original research, the conclusions will require further verification using high-quality randomized controlled trials.

**Systematic Review Registration:**

PROSPERO, Identifier: CRD42023401963.

## Introduction

Physical fitness (PF) is a valuable health marker. Maintaining satisfactory PF status in children and adolescents reduces the risk of obesity (1), cardiovascular disease (2), and diabetes (3) in adulthood. Indeed, approximately 80% of children and adolescents suffer from these health conditions because they are engaged in an insufficient level of physical activity (PA) (4–6). As a result, exercise intervention and PF promotion among children and adolescents have become a major focus of public health research. Schools are favorable setting for PF promotion (7) because they offer this age group a high amount active time during the school day (8). The school environment provides an equitable sociocultural environment for exercise behavior using various PF promotion programs (9). Studies have reported the impact of particular school-based interventions on health-related PF (10, 11). A recent systematic review found that school-based neuromuscular training is effective at increasing strength (12). Two systematic reviews with meta-analyses involving 11 and 35 randomized controlled trials (RCTs) found that high-intensity interval training (HIIT) in school contributes to greater improvement in muscular and aerobic fitness (13, 14). However, these pairwise meta-analyses only compared one type of exercise to regular physical education (PE) lessons. While one meta-analysis attempted to evaluate the impact of school-based PF promotion programs on obesity prevention (15), it still failed to assess the superiority of the diverse exercise types. Overall, the evidence needed to measure the comparative effectiveness of multiple school-based exercise treatments on PF remains limited.

Recently, gamified exercises with higher attractiveness have been embedded in school-based PA programs to enhance students’ enjoyment (16). One meta-analysis reported favorable results for active video games (AVGs) on body mass index (BMI) reduction in children and adolescents (17). Likewise, game-based exercise (GB) including small-side ball games in recreational sports and aerobic exercise performed with game patterns, attracts researchers’ attention. A previous review found that small-side football ball games had a similar effect as interval running on PF (18). However, whether these novel exercise programs are superior to other modalities remains unclear. Thus, AVGs and GB conducted in school were included in the comparison.

No systematic review has integrated and assessed the effects of various exercise treatments on anthropometry and body composition, muscular fitness (MF), and cardiorespiratory fitness (CRF) outcomes, concentrating exclusively among children and adolescents in school-based settings. This review used network meta-analysis (NMA), a newly recommended analysis tool in the field of PA and health promotion (19), to (1) evaluate the comparative efficacy of six exercise treatments performed in the school environment on anthropometry and body composition, MF and CRF and (2) construct an effectiveness hierarchy. Unlike pairwise meta-analysis, NMA is able to comparative multiple interventions as an intermediary for indirect comparisons even in the absence of direct comparative evidence. NMA also ranks probable success of each intervention.

## Methods

The study follows the relevant PRISMA checklist (20). The study protocol was registered prospectively in PROSPERO (registration code: CRD42023401963).

### Search strategy

A comprehensive computerized search of the Web of Science, PubMed, SPORTDiscus, and Scopus database was performed from inception until February 2023. The retrieval strategy was conducted using the PICOS tool: (P) Population: children and adolescents; (I) Intervention: active video games (AVGs), game-based exercise (GB), high-intensity interval training (HIIT), aerobic training (AT), strength training (ST), and combined aerobic and strength training (CT; Table 1); (C) Comparator: regular physical activity or physical education; (O) Outcomes: anthropometry and body composition, muscular fitness (MF), and cardiorespiratory fitness (CRF); (S) Study type: randomized controlled trials (RCTs) or quasi-RCTs. The detailed search algorithms are shown in Supplementary Table S1. Reference lists of the included studies and previous reviews were scanned for articles that met the eligibility criteria.

**Table 1:** Description of the exercise modes.

Exercise types	Description
AVGs	Frequency: 1–3 times/week, 20–30 min/session
	Intensity: light-to-moderate intensity
	Duration: ≥4 weeks
	Type: various commercial exergames (e.g., Xbox Kinect, Wii sports, Rhythmic Dance Games, and PlayStation)
GB	Frequency: 1–3 times/week, 15–25 min/session
	Intensity: moderate-to-vigorous intensity
	Duration: ≥4 weeks
	Type: small-side ball games in recreational sports and moderate intensive aerobic exercise performed with a game pattern
HIIT	Frequency: 2–3 times/week, 10–15 min/session
	Intensity: >75% VO_2max_ or > 80% HRmax
	Duration: ≥4 weeks
	Type: any type of interval training
ST	Frequency:2–3 times/week, 20–30 min/session
	Intensity: ≥50% 1RM
	Duration: ≥4 weeks
	Type: any form of strength training (e.g., bodyweight, free weights, and functional strength training)
AT	Frequency: 2–3 times/week, 20–30 min/session
	Intensity: >45% VO_2max_ or > 65% HRmax
	Duration: ≥4 weeks
	Type: any continuous aerobic training (e.g., running, walking, and cycling)
CT	A combination of CET and RT and concurrent training
CON	Regular physical activity or physical education course

### Eligibility criteria

The inclusion criteria for this systematic review and NMA were as follows: (1) peer-reviewed original research with full text in English over the past 20 years (January 2003 to February 2023); (2) study participants were children and adolescents aged 4–18 years of age enrolled in full-time or part-time education; (3) at least one exercise type, including AVGs, GB, HIIT, AT, ST, or CT, was employed in a school setting (intra-PE, or extra-PE during school hours); (4) anthropometry and body composition [body mass index (BMI), body fat percent (BF%), and waist circumference (WC)], MF [standing long jump (SLJ), countermovement jump (CMJ), push-ups, and 20-meter sprint (20-m sprint)], and/or CRF (shuttle running (SR), and VO_2max_) were used as outcomes; and (5) the intervention lasted at least 4 weeks. Both RCTs and quasi-RCTs were included given the difficulty of implementing RCTs in school settings. The inclusion of only RCTs may have led to the omission of relevant information. Participants with injuries or chronic or acute diseases, participants who were youth athletes, reviews and meta-analyses, studies lacking the required outcomes, or studies that were unable to identify implementation setting were excluded.

### Study selection and data extraction

Two independent authors (JW and YY) screened the literature based on the inclusion and exclusion criteria and read the full text to assess their eligibility. Any disagreements were handled by adjudications from other team members. The following data were extracted from eligible articles and recorded in EXCEL: (1) first author and publication year; (2) participant demographics (e.g., sample size, sex, and mean age); (3) intervention characteristics (exercise type, duration frequency, time in school); and (4) outcomes. EndNote X9 was used to consolidate and remove duplicates. If the same trial was published more than once, the most recent or more complete study was selected.

### Risk of bias assessment

Two authors (JW and YY) independently assessed the risk of bias (ROB) using the following seven dimensions from the Cochrane Handbook for Systematic Reviews of Interventions Version 5.1.0 tool (21): (1) randomized sequence generation; (2) treatment allocation concealment; (3) blinding of participants; (4) blinding of personnel; (5) incomplete outcome data; (6) selective reporting; and (7) other sources of bias. Eligible studies were divided into high risk (≥4), medium risk (3), and low risk (≤2) of bias based on the frequency of high-risk items. By default, all studies were classified as high ROB in the “blinding of participants” dimension since it is difficult to achieve participant blinding during exercise intervention protocols in school settings. Thus, this domain was not counted toward the overall score.

### Data synthesis and statistical analysis

All outcomes involved in this study were continuous variables, so the mean and standard deviations (SDs) were extracted from the included studies. Mean differences (MDs) were obtained by directly extracting or subtracting the mean at the post-training vs. pre-training. The unreported standard deviation difference was imputed according to the formula provided in the Cochrane Handbook (Supplementary Figure S37) (22). Standardized mean differences (SMDs) were applied when different evaluation methods or scales were used to measure the same indicator. When multiple posttests (e.g., multiple follow-ups) occurred, data measured immediately after the intervention were extracted. If multiple variations of the same interventions or different population subgroups were compared in an included study, the respective outcomes were pooled using the formula provided in Supplementary Figure S37 (22).

Statistical analyses were conducted using STATA 16.0 software. Heterogeneity was assessed using I^2^ statistic. Values of I^2^ ≤ 25, 25% < I^2^ ≤ 50, 50% < I^2^ ≤ 75%, and I^2^ > 75% represented no significant heterogeneity, low heterogeneity, medium heterogeneity, and high heterogeneity, respectively. A random-effects frequentist framework-based NMA was used to calculate pooled estimates and 95% confidence intervals (CI) (23). Network plots were created to visually demonstrate the geometry of various treatments. Each node corresponded to a certain treatment, and the node size represented the sample size. The lines linking the nodes indicated the direct head-to-head comparisons between interventions, and the line thickness between nodes represented the number of included studies. The Wald test and node splitting methods were used to evaluate global and local inconsistencies, respectively. The surface under the cumulative ranking curve (SUCRA) shows the ranking probability of each intervention. The larger the SUCRA value, which ranges from 0 to 100%, the more significant the intervention effect. Network funnel plots were generated to identify whether publication bias was caused by any small sample studies. To examine robustness, sensitivity analyses were performed by eliminating individual studies separately to evaluate the impact of each study on the overall heterogeneity.

## Results

### Literature search and selection

**Figure 1:**
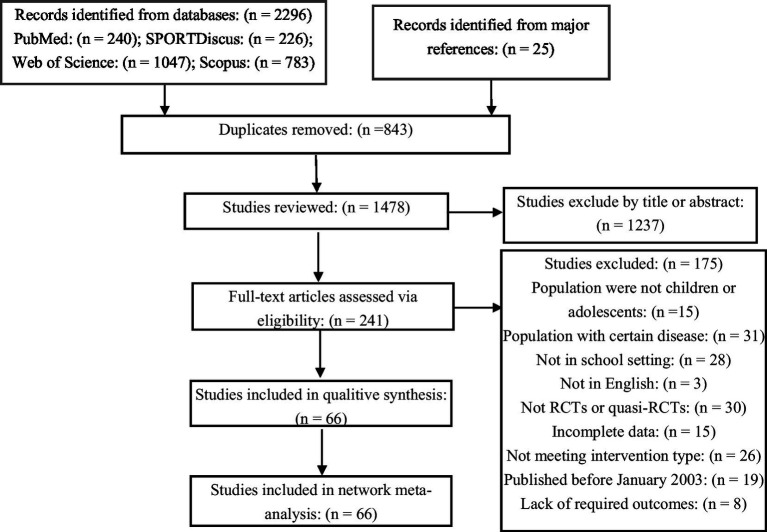
Flowchart of study selection.

A total of 2,296 studies were obtained by a preliminary search of the databases, and an additional 25 studies were obtained from existing systematic reviews. After removing duplicate studies and screening titles and abstracts, 241 studies were carefully read, of which 66 studies were finally included. A flowchart of the selection process is shown in Figure 1.

### Description of the included studies

Basic information about the eligible studies is shown in Supplementary Table S3. These included studies were conducted in North America (*n* = 3), South America (*n* = 3), Europe (*n* = 38), Asia (*n* = 13), Oceania (*n* = 7), and Africa (*n* = 2). A total of 8,578 children and adolescents (48% girls) with a mean age of 13.6 ± 4.3 were included in the 66 studies. Nine studies recruited only girls, eight studies recruited only boys, and the remaining 49 recruited both boys and girls. Fourteen studies focused on overweight or obese children and adolescents.

A total of 3,486 participants (1,685 girls) were included in the control group (CON) group that participated in regular physical activity (PA) or physical education (PE), 426 participants (171 girls) were included in the active video games (AVGs) group (24–31), 1,670 participants (803 girls) were included in the game-based exercise (GB) group (32–42), 1,085 participants (522 girls) were included in the high-intensity interval training (HIIT) group (36, 37, 43–65), 1,005 participants (462 girls) were included in the strength training (ST) group (41, 66–82), 599 participants (300 girls) were included in the aerobic training (AT) group (49, 53, 56, 61, 62, 66, 76, 83–87), and 307 participants (197 girls) were included in the combined aerobic and strength training (CT) group (68–71, 76, 88, 89). Interventions lasted on average of 13.9 ± 9.6 weeks and ranged from 4 to 50 weeks, with 73% of studies lasting less than 12 weeks. The average number of sessions per week was 2.7 ± 0.8. Twenty-three interventions were conducted intra-PE, 38 were conducted extra-PE, and five occurred both intra- and extra-PE.

### Risk of bias assessment results

A summary of risk of bias (ROB) evaluation results is shown in Supplementary Table S2. Ten studies had high a ROB, 38 had a moderate ROB, and the remaining 18 had a low ROB. For each individual ROB item, 48 studies had a high random sequence generation. Only two studies demonstrated a low ROB in allocation concealment, while four mentioned blinding of research personnel. Seven studies had a high ROB due to incomplete outcome data and 36 studies had unclear ROB from selective reporting. Five studies had a high risk of other bias. Table 2 presents the ROB results in each intervention.

**Table 2:** ROB in each intervention.

Exercise types	Low risk	Moderate risk	High risk
AVGs	4	1	3
GB	4	6	1
HIIT	5	17	2
ST	5	10	3
AT	3	8	1
CT	1	4	2

Each cell represents the number of included studies. AT, aerobic training; HIIT, high-intensity interval training; AVGs, active video games; GB, game-based exercise; CT, combined aerobic and strength training; ST, strength training.

### Network meta-analysis

**Figure 2:**
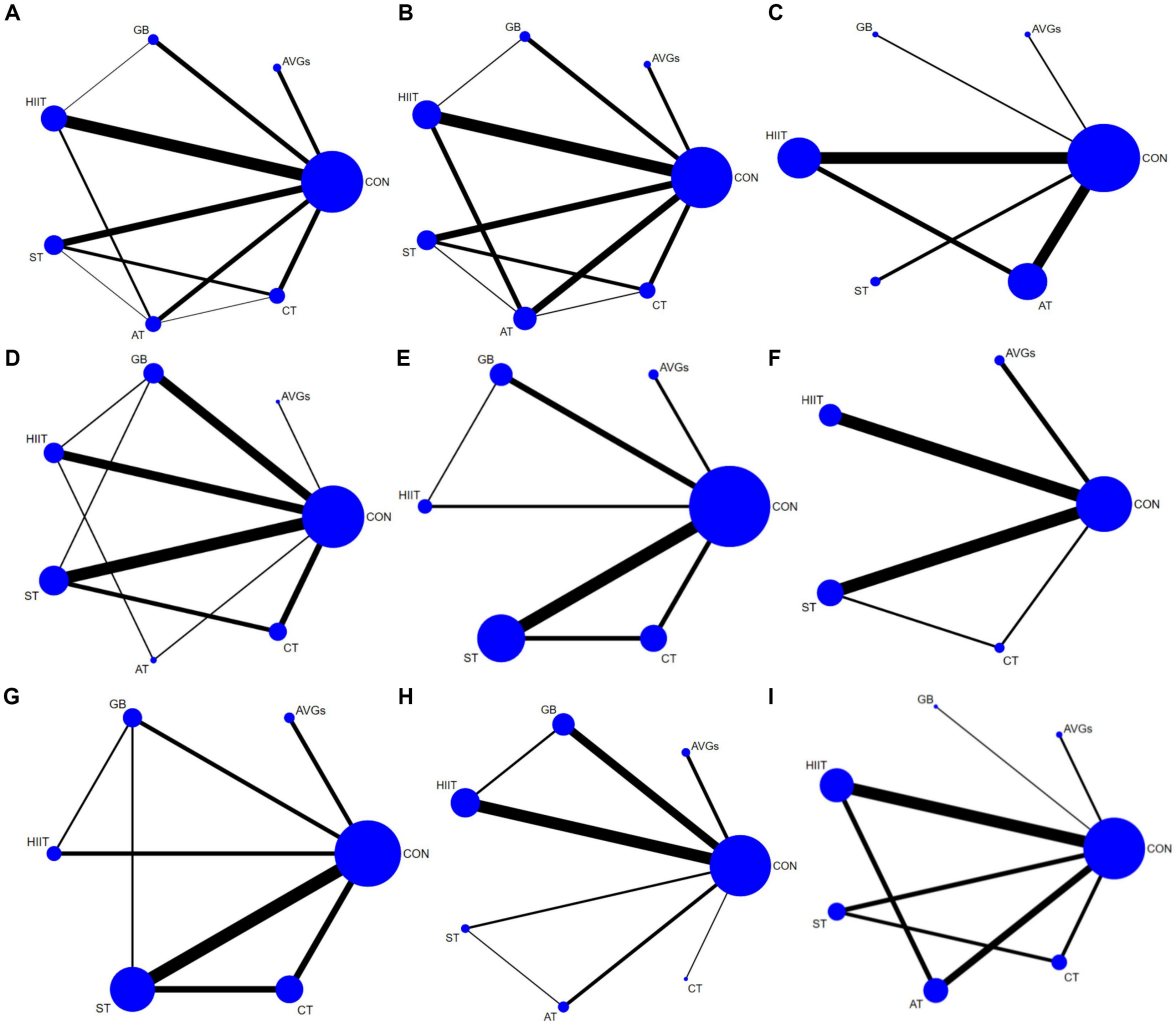
Network plots for outcome comparisons. The node size represented the sample size corresponding to the intervention, and the line thickness between nodes represented the number of included studies. **(A)** BMI, **(B)** body fat percent, **(C)** waist circumference, **(D)** standing long jump, **(E)** countermovement jump, **(F)** push-ups, **(G)** 20-m sprint, **(H)** shuttle running, and **(I)** VO_2max_.

**Figure 3:**
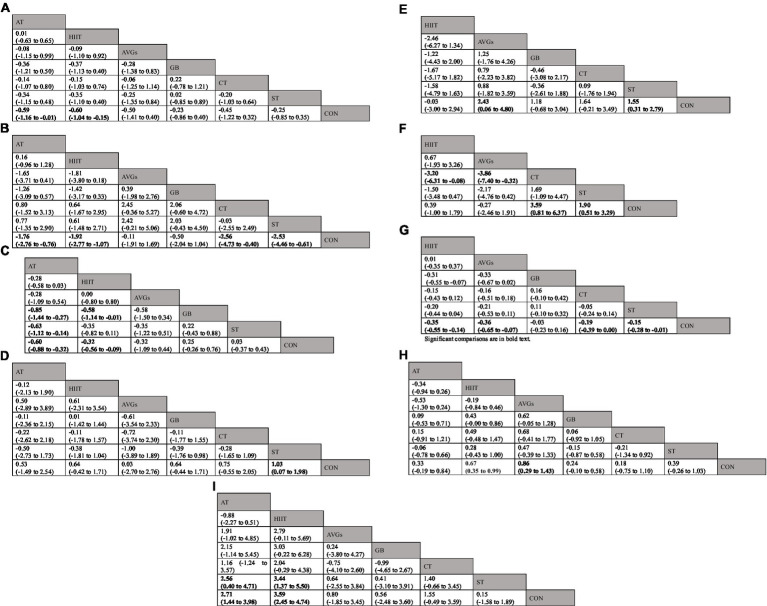
League table of comparative effectiveness results for **(A)** BMI [kg·m^−2^], **(B)** body fat percent [%], **(C)** waist circumference, **(D)** standing long jump, **(E)** countermovement jump [cm], **(F)** push-ups, **(G)** 20-m sprint [s], **(H)** shuttle running, **(I)** VO_2max_ [mL·kg^−1^·min^−1^]. Significant comparisons are in bold text. Each cell presents an SMD/MD with a 95%CI. For **A–C,G**, lower negative SMD/MD values indicate positive effects on PF. Thus, a negative SMD/MD supports the upper-left intervention. For **D–F,H,I**, higher positive SMD/MD values indicate positive effects on PF. Thus, a positive SMD/MD supports the upper-left intervention, and a negative SMD/MD supports the lower-right intervention. 95%CI = 95%confidence interval; AT, aerobic training; HIIT, high-intensity interval training; AVGs, active video games; GB, game-based exercise; CT, combined aerobic and strength training; ST, strength training; CON, control group; SMD, standardized mean difference; MD, mean difference.

Three anthropometry and body composition [body mass index (BMI; kg·m^−2^), waist circumference (WC), and body fat percent (BF; %) measurements, four muscular fitness (standing long jump (SLJ), countermovement jump (CMJ; cm), push-up, and 20-meter sprint (20-m sprint; s)] measurements, and two cardiorespiratory fitness [shuttle running (SR), VO_2max_; mL·kg^−1^·min^−1^)] measurements were included in the network meta-analysis (NMA). The geometry of different interventions for each outcome is shown in Figure 2. Each node represents one type of exercise treatment, while node size represents the sample size used in the intervention. The lines between two nodes indicate direct comparisons between the exercise types, with thicker lines indicating that more studies were included. Additional information about the contribution plots of comparative evidence is available in Supplementary Figures S1–S9. Global inconsistency testing was not significant (all Wald test statistic *p* values >0.5). Homoplastically, the results of local inconsistency testing using the node splitting method showed that each direct and indirect comparison among estimates was coincident for all outcomes (all *p*-values > 0.5; Supplementary Tables S4–S12). Forest plots with 95% CI are displayed in Supplementary Figures S10–S18. The comparative effects of each exercise type is shown in Figures 3A–I. Funnel plots evaluating publication bias are available in Supplementary Figures S19–S27. Supplementary Figures S28–S36 describes the surface under the cumulative ranking curve for all interventions. Larger SUCRA values, represent a higher probability that a treatment will be effective. Using the SUCRA value and mean rank of all exercise types were sorted (Table 3). Besides, due to the limitation of original studies, hand grip strength and sit-ups were not able to form a complete loop in NMA. Therefore, they were excluded.

**Table 3:** Rankings for six exercise types in order of effectiveness.

	SUCRA, %	Mean rank		SUCRA, %	Mean rank		SUCRA, %	Mean rank
Body mass index			Body fat percent			Waist circumstance		
AVGs	60.3	3.4	AVGs	18.0	5.9	AVGs	64.4	2.8
GB	37.8	4.7	GB	27.1	5.4	GB	11.0	5.5
HIIT	72.6	2.6	HIIT	68.6	2.9	HIIT	68.9	2.6
ST	38.8	4.7	ST	80.9	2.1	ST	29.8	4.5
AT	70.4	2.8	AT	62.2	3.3	AT	94.2	1.3
CT	57.5	3.5	CT	81.0	2.1	CON	31.8	4.4
CON	12.5	6.2	CON	12.2	6.3			
Standing long jump			Countermovement jump			Push-ups		
AVGs	35.6	5.6	AVGs	81.9	1.9	AVGs	19.5	4.2
GB	55.1	4.7	GB	52.6	3.4	HIIT	37.5	3.5
HIIT	54.9	3.6	HIIT	23.4	4.8	ST	74.6	2.0
ST	74.6	3.7	ST	63.5	2.8	CT	96.0	1.2
AT	50.3	2.5	CT	65.1	2.7	CON	22.3	4.1
CT	59.6	4.6	CON	13.5	5.3			
CON	19.7	3.4						
20-m sprint			Shuttle running			VO_2max_		
AVGs	84.0	1.8	AVGs	89.1	1.7	AVGs	40.3	4.6
GB	18.7	5.1	GB	37.8	4.7	GB	35.4	4.9
HIIT	85.6	1.7	HIIT	78.9	2.3	HIIT	96.5	1.2
ST	45.8	3.7	ST	52.1	3.9	ST	23.4	5.6
CT	57.5	3.1	AT	46.4	4.2	AT	78.9	2.3
CON	8.4	5.6	CT	34.5	4.9	CT	56.7	3.6
			CON	11.0	6.3	CON	18.9	5.9

Higher SUCRA and lower mean ranks indicate better-performed interventions. AT, aerobic training; HIIT, high-intensity interval training; AVGs, active video games; GB, game-based exercise; CT, combined aerobic and strength training; ST, strength training; CON, control group; SUCRA, surface under the cumulative ranking curve.

### Anthropometry and body composition outcomes

#### Body mass index

Forty studies involving 4,841 participants and all six exercise types reported BMI. Overall effects indicated that school-based exercise interventions can effectively reduce BMI (MD = −0.39 kg·m^−2^, 95% CI = −0.62 to −0.15, *p* = 0.001). Compared with CON, AT (MD = −0.59 kg·m^−2^, 95% CI = −1.16 to −0.01, *p* = 0.043) and HIIT (MD = −0.60 kg·m^−2^, 95% CI = −1.04 to −0.15, *p* = 0.009) significantly reduced BMI while AVGs (MD = −0.50 kg·m^−2^, 95% CI = −1.41 to 0.40, *p* = 0.275), GB (MD = −0.23 kg·m^−2^, 95% CI = −0.86 to 0.40, *p* = 0.475), ST (MD = −0.25 kg·m^−2^, 95% CI = −0.85 to 0.35, *p* = 0.418), and CT (MD = −0.45 kg·m^−2^, 95% CI = −1.22 to 0.32, *p* = 0.255) had no significant impact on BMI (Figure 3A; Supplementary Figure S10). HIIT had the highest probability (SUCRA = 72.6%) of being the most treatment for lowering BMI (Table 3; Supplementary Figure S28).

#### Body fat percentage

Thirty studies involving 3,332 participants and all six exercise types included BF%. Overall effects indicated that school-based exercise interventions can effectively reduce BF% (MD = −1.78%, 95% CI = −2.38 to −1.17, *p* < 0.001). Compared with CON, CT (MD = −2.56%, 95% CI = −4.73 to −0.40, *p* = 0.022), ST (MD = −2.53%, 95% CI = −4.46 to −0.61, *p* = 0.011), HIIT (MD = −1.92%, 95% CI = −2.77 to −1.07, *p* < 0.001), and AT (MD = −1.76%, 95% CI = −2.76 to −0.76, *p* < 0.001) significantly reduced BF% (Figure 3B; Supplementary Figure S11). CT was the most effective intervention for BF% reduction (SUCRA = 81.0%; Table 3; Supplementary Figure S29). Compared with CON, AVGs (MD = −0.11%, 95% CI = −1.91 to 1.69, *p* = 0.909), and GB (MD = −0.50%, 95% CI = −2.04 to 1.04, *p* = 0.534) had no significant impact on BF% (Figure 3B; Supplementary Figure S11).

#### Waist circumference

Sixteen studies involving 1,073 participants and five exercise modalities in addition to CT explored the effects of exercise on waist circumference. Overall effects indicated that school-based exercise interventions can effectively reduce WC (SMD = −0.31, 95% CI = −0.52 to −0.10, *p* = 0.004). Compared with CON, HIIT (SMD = −0.32, 95% CI = −0.56 to −0.09, *p* = 0.007) and AT (SMD = −0.60, 95% CI = −0.88 to −0.32, *p* < 0.001) significantly reduced WC (Figure 3C; Supplementary Figure S12). AT had the highest probability (SUCRA = 94.2%) of reducing WC (SUCRA = 94.2%; Table 3; Supplementary Figure S30). Compared with CON, AVGs (SMD = −0.32, 95% CI = −1.09 to 0.44, *p* = 0.405), GB (SMD = 0.25, 95% CI = −0.26 to 0.76, *p* = 0.333), and ST (SMD = 0.03, 95% CI = −0.37 to 0.43, *p* = 0.887) had no significant impact on WC (Figure 3C; Supplementary Figure S12).

### Muscular fitness outcomes

#### Standing long jump

Twenty-two studies involving 4,185 participants and all six interventions reported the effects of multiple exercise modes on SLJ. Overall effects indicated that school-based exercise interventions can effectively improve SLJ (SMD = 0.63, 95% CI = 0.38 to 0.89, *p* < 0.001). Compared with CON, ST (SMD = 1.03, 95% CI = 0.07 to 1.98, *p* = 0.035) significantly improved SLJ while AVGs (SMD = 0.03, 95% CI = −2.70 to 2.76, *p* = 0.983), GB (SMD = 0.64, 95% CI = −0.44 to 1.71, *p* = 0.245), HIIT (SMD = 0.64, 95% CI = −0.42 to 1.71, *p* = 0.234), AT (SMD = 0.53, 95% CI = −1.49 to 2.54, *p* = 0.607), and CT (SMD = 0.75, 95% CI = −0.55 to 2.05, *p* = 0.258) had no significant impact on SLJ (Figure 3D; Supplementary Figure S13). ST had the highest probability of improving SLJ (SUCRA = 74.6%; Table 3; Supplementary Figure S31).

#### Countermovement jump

Fifteen studies involving 934 participants and five exercise modalities other than AT examined the effects of various exercise treatments on CMJ. Overall effects indicated that school-based exercise interventions can effectively improve CMJ (MD = 1.22 cm, 95% CI = 0.27 to 2.18, *p* = 0.012). Compared with CON, AVGs (MD = 2.43 cm, 95% CI = 0.06 to 4.80, *p* = 0.041) and ST (MD = 1.55 cm, 95% CI = 0.31 to 2.79, *p* = 0.014) significantly improved CMJ while GB (MD = 1.18 cm, 95% CI = −0.68 to 3.04, *p* = 0.212), HIIT (MD = −0.03 cm, 95% CI = –3.00 to 2.94, *p* = 0.983), and CT (MD = 1.64 cm, 95% CI = −0.21 to 3.49, *p* = 0.083) had no significant impact on CMJ (Figure 3E; Supplementary Figure S14). AVGs had the highest probability of improving CMJ (SUCRA = 81.9%; Table 3; Supplementary Figure S32).

#### Push-ups

Twelve studies involving 1,557 participants and four interventions in addition to AT and GB assessed the effects of diverse exercise regimens on push-up ability. Overall effects indicated that school-based exercise interventions can effectively improve push-up ability (SMD = 1.14, 95% CI = 0.35 to 1.93, *p* = 0.005). Compared with CON, CT (SMD = 3.59, 95% CI = 0.81 to 6.37, *p* = 0.012) and ST (SMD = 1.90, 95% CI = 0.51 to 3.29, *p* = 0.007) significantly improved push-up ability while AVGs (SMD = −0.27, 95% CI = −2.46 to 1.91, *p* = 0.806) and HIIT (SMD = 0.39, 95% CI = −1.00 to 1.79, *p* = 0.581) had no significant impact on SLJ (Figure 3F; Supplementary Figure S15). CT had the highest probability of improving push-up ability (SUCRA = 96.0%; Table 3; Supplementary Figure S33).

#### 20-m sprint

Ten studies involving 1,059 participants reported the effects of five exercise modalities on 20-m sprint performance. Overall effects indicated that school-based exercise interventions can effectively improve 20-m sprint performance (MD = −0.17 s, 95% CI = −0.24 to −0.10, *p* < 0.001). Compared with CON, HIIT (MD = −0.35 s, 95% CI = −0.55 to −0.14, *p* = 0.001), AVGs (MD = −0.36 s, 95% CI = −0.65 to −0.07, *p* = 0.016), ST (MD = −0.15 s, 95% CI = −0.28 to −0.01, *p* = 0.035), and CT (MD = −0.19 s, 95% CI = −0.39 to 0.00, *p* = 0.047) significantly advanced the 20-m sprint performance while GB (MD = −0.03 s, 95% CI = −0.23 to 0.16, *p* = 0.729) had no significant impact on 20-m sprint performance (Figure 3G; Supplementary Figure S16). HIIT had the highest possibility of improving participant 20-m sprint ability (SUCRA = 85.6%; Table 3; Supplementary Figure S34).

### Cardiorespiratory fitness outcomes

#### Shuttle running

Twenty-three studies involving 3,927 participants explored the impact of all six exercise types on SR. Overall effects indicated that school-based exercise interventions can effectively improve SR performance (SMD = 0.44, 95% CI = 0.29 to 0.59, *p* < 0.001). Compared with CON, AVGs (SMD = 0.86, 95% CI = 0.29 to 1.43, *p* = 0.003) and HIIT (SMD = 0.67, 95% CI = 0.35 to 0.99 *p* < 0.001) significantly advanced SR while GB (SMD = 0.24, 95% CI = −0.10 to 0.58, *p* = 0.170), ST (SMD = 0.39, 95% CI = −0.26 to 1.03, *p* = 0.238), AT (SMD = 0.33, 95% CI = −0.19 to 0.84, *p* = 0.210), and CT (SMD = 0.18, 95% CI = −0.75 to 1.10, *p* = 0.710) had no significant impact on (Figure 3H; Supplementary Figure S17). AVGs had the highest probability of improving SR (SUCRA = 89.1%; Table 3; Supplementary Figure S35).

#### Maximum oxygen uptake (VO_2max_)

Twenty-one studies involving 2,842 participants reported the effects of all six treatments on VO_2max_. Overall effects indicated that school-based exercise interventions can effectively improve VO_2max_ (MD = 2.50 mL·kg^−1^·min^−1^, 95% CI = 1.78 to 3.22, *p* < 0.001). Compared with CON, HIIT (MD = 3.59 mL·kg^−1^·min^−1^, 95% CI = 2.45 to 4.74, *p* < 0.001) and AT (MD = 2.71 mL·kg^−1^·min^−1^, 95% CI = 1.44 to 3.98, *p* < 0.001) significantly improved VO_2max_ while AVGs (MD = 0.80 mL·kg^−1^·min^−1^, 95% CI = −1.85 to 3.45, *p* = 0.428), GB (MD = 0.56 mL·kg^−1^·min^−1^, 95% CI = −2.48 to 3.60, *p* = 0.669), ST (MD = 0.15 mL·kg^−1^·min^−1^, 95% CI = −1.58 to 1.89, *p* = 0.370), and CT (MD = 1.55 mL·kg^−1^·min^−1^, 95% CI = −0.49 to 3.59, *p* = 0.054) had no significant impact on VO_2max_ (Figure 3I; Supplementary Figure S18). Overall, HIIT had the highest probability of increasing VO_2max_ (SUCRA = 96.5%; Table 3; Supplementary Figure S36).

## Discussion

To the best of our current knowledge, it is the first network meta-analysis (NMA) to compare the effects of school-based exercise modalities for physical fitness (PF) promotion among young students. High-intensity interval training (HIIT) was the most effective intervention reducing body mass index (BMI), and elevating VO_2max_, and 20-meter sprint (20-m sprint) performance. Aerobic training (AT) had the highest probability of reducing waist circumference (WC) while active video games (AVGs) emerged as a promising modality for improving countermovement jump (CMJ)and shuttle running (SR). Strength training (ST) was the best exercise for standing long jump (SLJ). Of the six interventions discussed, combined aerobic and strength training (CT) was most effective at lowering body fat percent (BF%) and increasing push-up repetitions.

### Anthropometry and body composition

BMI, total body fat, and abdominal adiposity are important predictors of cardiometabolic risk among youth. A recent systematic review used pairwise meta-analysis to assess the effects of HIIT (MD = −1.66 kg·m^−2^) and moderate-intensity continuous training (MD = −2.37 kg·m^−2^) on pediatric BMI and found no significant differences between the two exercises (90). While these findings were similar to those reported by the current study, the effects provided by this NMA were smaller, possibly due to differences in participant demographics. While the review focused exclusively on obese children, the current study did not make a strict distinction between overweight and normal weight participants. Exercise tends to have a greater impact on the body composition of obese or overweight children than those of normal weight (91).

One study found that exercise had a greater impact on BF% than BMI (92). This may be because a decrease in BMI can correlate with both weight loss and an increase in height caused by the natural growth of children and adolescents. In addition, because it is the most metabolically hazardous tissue, body fat serves as a valuable health measure of exercise intervention assessments. However, there is limited evidence showing that school-based exercise programs can reduce student BF%. A recent meta-analysis summarized the effect of a school-based HIIT program on BF% and found a pooled effect size of −1.7% (13). The current NMA went one step further by evaluating different exercise types. In addition to HIIT, AT, ST, and CT also lowered BF% of children and adolescents, a finding consistent with a previous NMA that compared the effects of five exercise types on the PF of adults (93). Although the current NMA found that CT was the best exercise mode for reducing body fat, HIIT was more efficient. Additional research is needed to compare the effect of CT and HIIT on fat loss.

In addition to total body fat, abdominal adiposity correlates closely with all-cause mortality (94). WC is often used as an indicator of abdominal adiposity and serves as a warning of potential health risks. For example, the risk of cardiovascular disease increases by 2% for each 1 cm increase in WC (95). While prior studies have suggested that HIIT does not significantly decrease WC in adolescents (96), more recent evidence contradicts these results (97). Similarly, this study found that HIIT and AT were effective at reducing WC in youth.

Surprisingly, while prior studies showed that AVGs significantly reduced BMI and BF% in children and adolescents, the current study found that school-based AVGs did not have a significant impact on participant anthropometry and body composition (98, 99). Differences in environmental factors may explain this inconsistency. While the articles included in this NMA study were exclusively conducted in schools, other reviews incorporated home or laboratory settings that were more beneficial to AVGs programs. Thus, more high-quality RCTs are needed to explore the influences of school-based AVGs on anthropometry and body composition.

### Muscular fitness

Maximum muscular strength, muscular power, and endurance are potentially correlated with cardiovascular variables and the future health of children and adolescents (100). One prior review found that school-based interventions have a small-to-moderate effect on muscular fitness (101), which is congruent with the results of the current study. However, distinct from the strength exercise guidelines (102), this NMA suggested that ST had a relatively small impact on muscular fitness (MF). The development of muscle strength involves both neuromuscular adaptation and muscle hypertrophy. Thus, to improve MF through ST, a detailed assessment of training prescription (i.e., frequency, load, volume, and duration) should be conducted. However, it can be difficult to develop in-school exercise programs that strictly following the prescription when there is a high number of students. In addition, there is variation in the efficacy of different ST modes. Compared with bodyweight training, for example, plyometric training has a greater impact on CMJ and SLJ (103). Unfortunately, the use of specialized plyometric training in school settings was not assessed in this review. Some teachers also oppose school-based ST due to their limited experience and qualifications or low confidence in the training plans (104), further limiting the benefits of this intervention.

Of note, the current NMA found that AVGs are an innovative exercise mode with the highest possibility of increasing vertical jump performance. Compared to regular physical activity (PA) or physical education (PE), AVGs led to statistically significant and clinically important changes in CMJ. Since the exercise intensity of most commercial AVGs (e.g., Xbox Kinect, Wii, PlayStation) cannot reach the minimum threshold needed to stimulate growth in muscle strength (27), it is probable that the increase in CMJ performance caused by AVGs may be related to elevated locomotor skills rather than muscle strength. Two previous reviews support this inference, revealing that AVGs had a small but statistically significant effects on the fundamental motor skills of young people (105, 106). However, the disadvantages of AVGs are obvious as well. Since the sensors embedded in exergames are unable to precisely track human movement, skill-specific motor learning cannot be guided. This explains why the effect size reported in these reviews was smaller than that observed in a previous meta-analysis of foundational motor skill (FMS) interventions presented in real-life situations (107).

Push-ups provide a simple and valid muscular endurance test for the upper body. Higher push-up capacity relates to a lower incidence of cardiovascular disease events (108). The current NMA found that CT was the most promising exercise treatment for enhancing push-ups. While this supports the findings of other studies, limited data have been collected in the school environment. One randomized controlled trial (RCT) compared the effects of a 22-week CT to ST and AT alone on obese adolescents, and CT was shown to have a superior impact on muscle endurance (109). The current study also found that HIIT had no effect on push-ups, which is inconsistent with previous reports. For example, Eather et al. reported 4.0 repetition (95% CI: 1.2 to 6.8) push-up changes in favor of a HIIT program in the university setting (110). This discrepancy may stem from differences in the HIIT content. Indeed, push-up capacity only increases when relevant upper body motions are involved in the HIIT prescription.

HIIT had the highest possibility of improving participant 20-m sprint performance. This finding generally coincides with previous studies on the effect of HIIT and ST on sprint running (111, 112). The main factors that contribute to sprint performance are anaerobic power and leg muscle strength. Recent studies suggest that specialized HIIT and ST are the key to modifying these factors (113, 114), with running-based HIIT and velocity-based ST showing relatively higher efficacy. Running-based HIIT employs stretch-shortening cycle (SSC) actions that involve the sequential assortment of eccentric and concentric muscle actions. SSC improves concentric power output, which increases maximal running speed (115). Velocity-based ST tends to improve strength and power via neural mechanisms (116).

### Cardiorespiratory fitness

CRF has a positive impact on physical and mental health (117) and academic performance (118) in teenagers, and physical exercise is recommended as a cost-effective tool to sustain CRF (119). The current study found that AVGs and HIIT were most effective at improving shuttle running and VO_2max_ respectively. The impact of AVGs was strongly impacted by the intervention arrangements used in each study. Due to governmental restrictions associated with the COVID-19 pandemic, regular daily PA was reduced for participants in the CON and their performance in shuttle running decreased significantly (26), leading to an unexpected gap between the experimental and CON groups. Without this interference, our calculations suggest that HIIT has a superior impact on shuttle running and VO_2max_, a result consistent with several pairwise meta-analyses (120–122). These improvements could be related to exercise-induced advantageous mitochondrial adaptations as well as an increase in blood capillarization, oxidative enzyme activity, and oxygen transport to the muscular system (123).

## Strengths and limitations

The strengths of this study include the following: (1) a considerable sample size (*n* = 8,578) of children and adolescents that provided enough power to identify statistically significant mean differences; (2) the incorporation of two emerging exercise interventions (AVGs and game-based exercise) that catered to the latest trends; and (3) the use of strict eligibility criteria to ensure that data were extracted from high-quality literature.

The study limitations are as follows: (1) low methodological quality of the included studies, with only 18 using random sequence generation methods and two mentioning whether allocation concealment was performed. Unclear allocation concealment may exaggerate study results and increase heterogeneity in meta-analysis; (2) the small number of AVGs studies may have reduced the robustness of the results and biased comparisons; (3) the failure to conduct detailed subgroup analysis for intra- and extra-PE interventions, making it difficult to explore the heterogeneity among studies.

## Conclusion

This systematic review using NMA suggested that except for GB, school-based exercise interventions were associated with an improvement in PF among children and adolescents. Based on these findings, we recommend integrating HIIT into PE classes and adding AT and ST to extracurricular activities. It is encouraged that exergaming systems be introduced into primary and secondary schools to improve student exercise enjoyment and PF. However, because the school-based exercise interventions evaluated by this study were applied to different populations (e.g., boys, girls, students of normal weight or overweight) and were largely affected by PE teachers, additional high quality RCTs are needed to explore the influence of teacher and student-related factors on the effectiveness of various exercise interventions.

## Author contributions

JW and YY contributed to study design, literature research, data extraction, risk of bias assessment, and drafting of the manuscript. HY and LL made substantial contributions to risk of bias assessment and data analysis. YC was a significant manuscript reviser. YS played important role in concept and design. All authors contributed to the article and approved the submitted version.

## Conflict of interest

The authors declare that the research was conducted in the absence of any commercial or financial relationships that could be construed as a potential conflict of interest.

## Publisher’s note

All claims expressed in this article are solely those of the authors and do not necessarily represent those of their affiliated organizations, or those of the publisher, the editors and the reviewers. Any product that may be evaluated in this article, or claim that may be made by its manufacturer, is not guaranteed or endorsed by the publisher.

## Glossary

PF: Physical fitness
PA: Physical activity
AVGs: Active video games
GB: Game-based exercise
HIIT: High-intensity interval training
ST: Strength training
AT: Aerobic training
CT: Combined aerobic and strength training
CON: Control group
RCTs: Randomized controlled trials
BMI: Body mass index
BF%: Body fat percent
WC: Waist circumference
SLJ: Standing long jump
CMJ: Countermovement jump
SR: Shuttle running
20-m sprint: 20-meter sprint
MF: Muscular fitness
CRF: Cardiorespiratory fitness
NMA: Network meta-analysis
PE: Physical education
