# Supplementary material for: Comparative effectiveness of school-based exercise interventions on physical fitness in children and adolescents: a systematic review and network meta-analysis
Source: Front Public Health. 2023 Jun 5;11:1194779. doi: 10.3389/fpubh.2023.1194779 (PMC10278967; doi:10.3389/fpubh.2023.1194779)
Supplement: Supplementary file 1 [file Table_1.DOC]

**Supporting Documentation**

Figure S1-S9: Contribution plots of direct and indirect evidence

Figure S10-S18: Forrest plot of eligible comparisons

Figure S19-S27: Funnel plot graphics

Figure S28-S36: SUCRA for each intervention

Figure S37: Cochrane Handbook Formulas

Table S1: Search strategy

Table S2: risk of bias results table

Table S3: Summary of included studies

Table S4-S12: Inconsistency result table

**Figure S1-S9:** Contribution plots of direct and indirect evidence in this network meta-analysis.

**Figure S1:** Contribution plots of direct and indirect evidence for BMI.


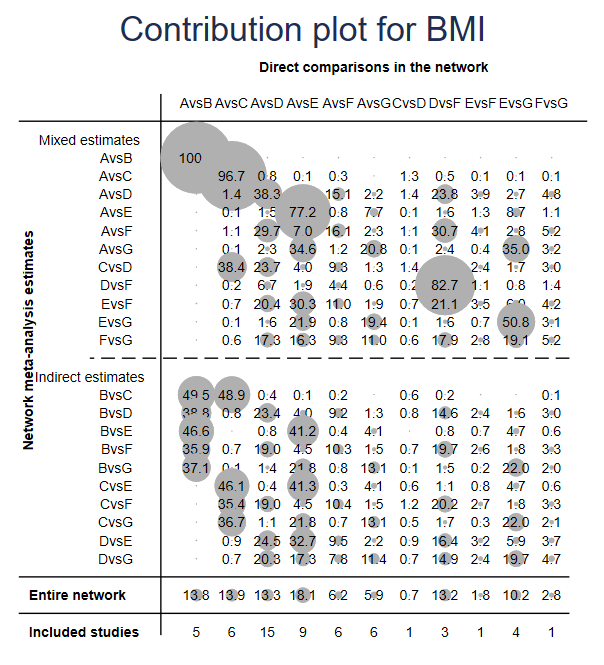


**Figure S2:** Contribution plots of direct and indirect evidence for body fat percent.

**
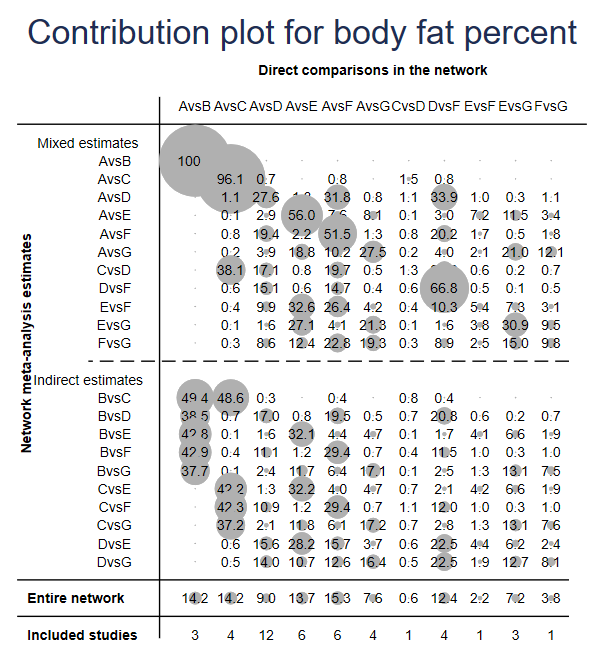
**

**Figure S3:** Contribution plots of direct and indirect evidence for waist circumstance.

**
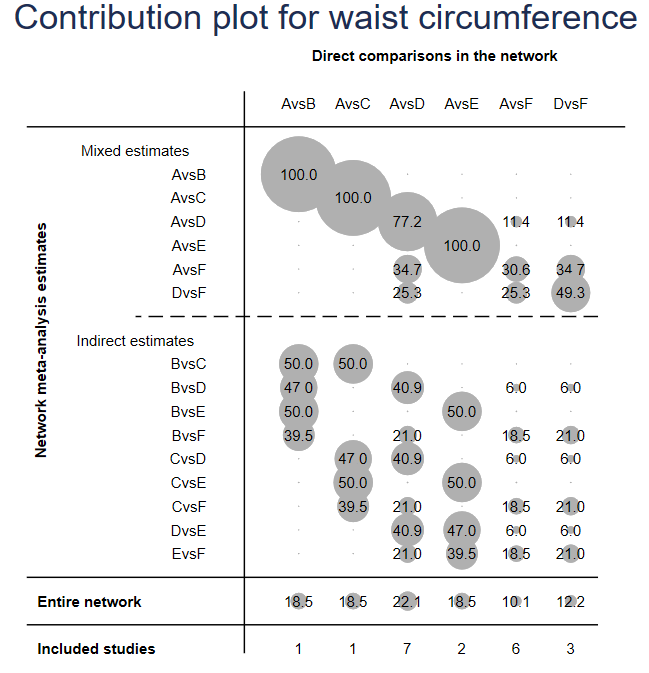
**

**Figure S4:** Contribution plots of direct and indirect evidence for standing long jump.

**
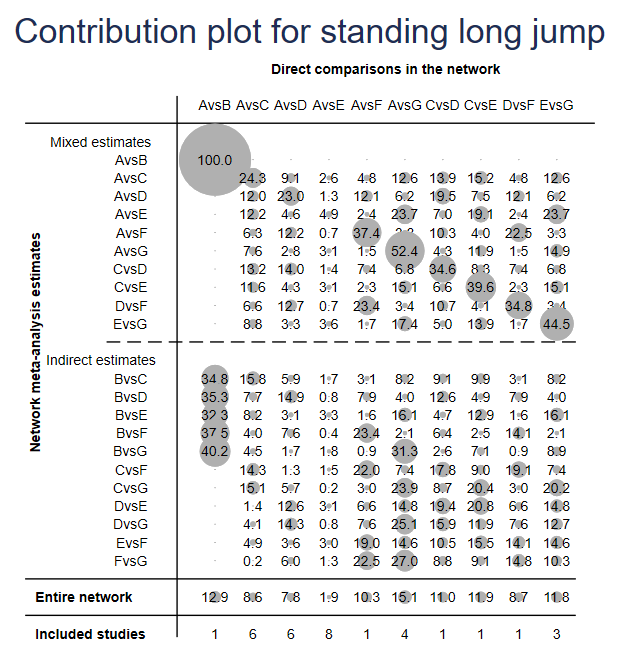
**

**Figure S5:** Contribution plots of direct and indirect evidence for countermovement jump
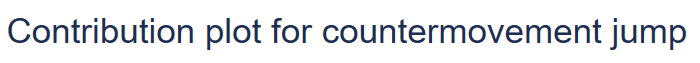
.

**
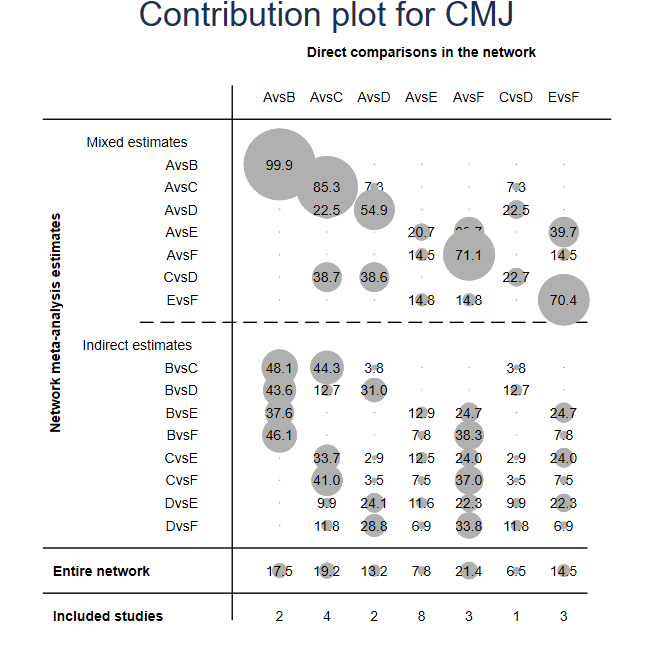
**

**Figure S6:** Contribution plots of direct and indirect evidence for push-ups.


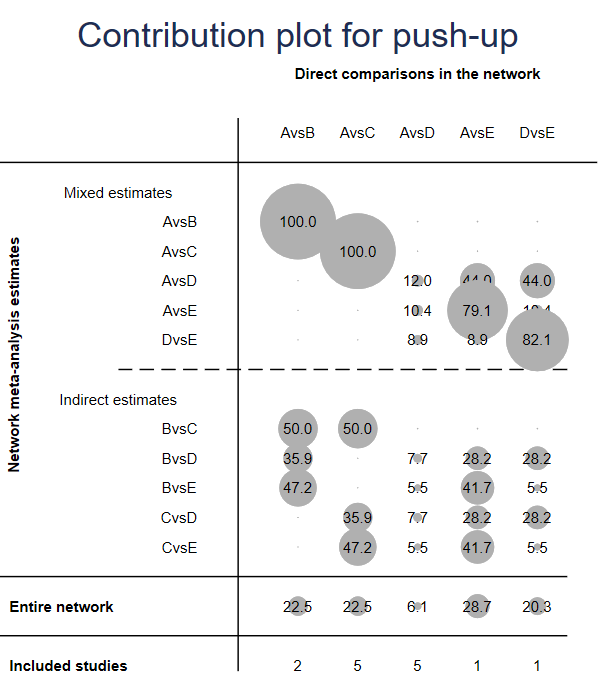


**Figure S7:** Contribution plots of direct and indirect evidence for 20-m sprint.

**
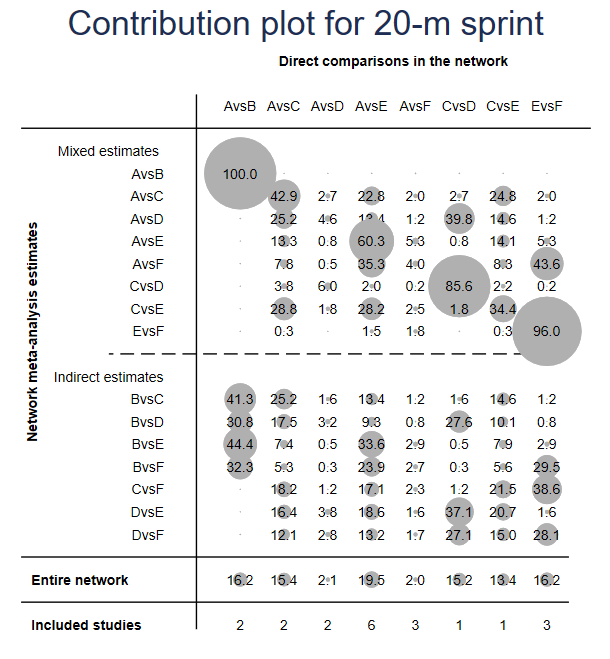
**

**Figure S8:** Contribution plots of direct and indirect evidence for shuttle running.


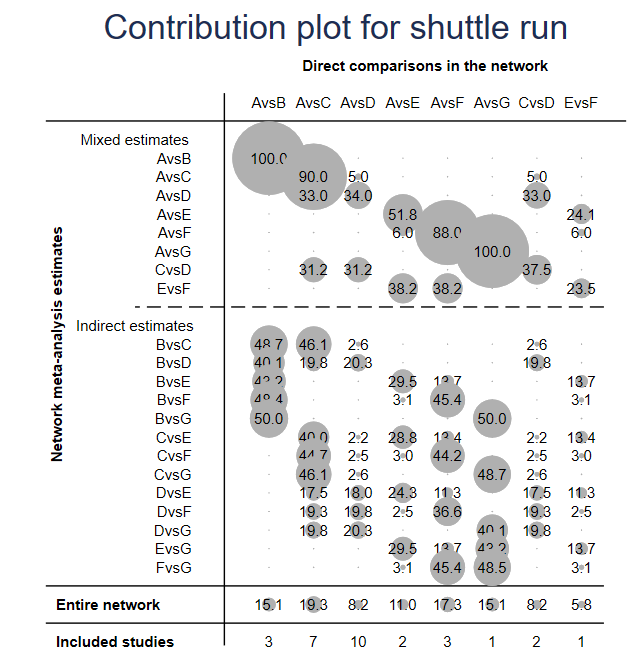


**Figure S9:** Contribution plots of direct and indirect evidence for VO_2max_.


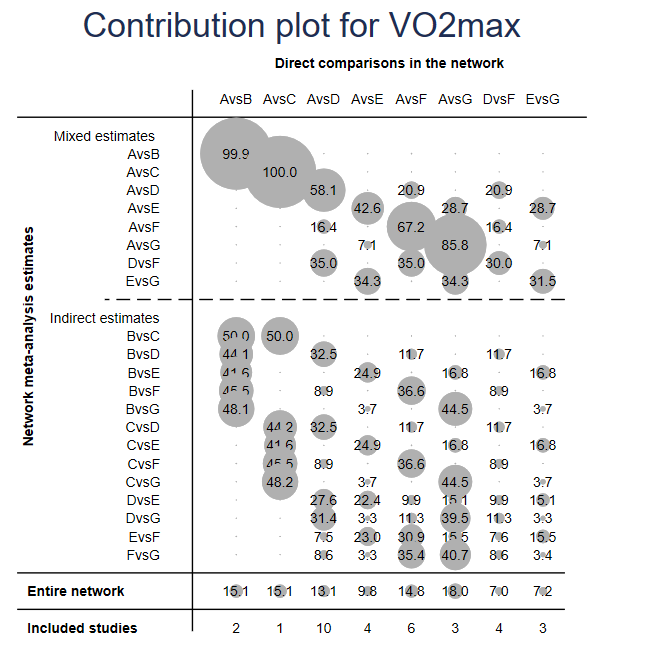


**Figure S10-S18:** Forrest plot of eligible comparisons in this network meta-analysis.

**Figure S10:** Forrest plot of eligible comparisons for BMI.


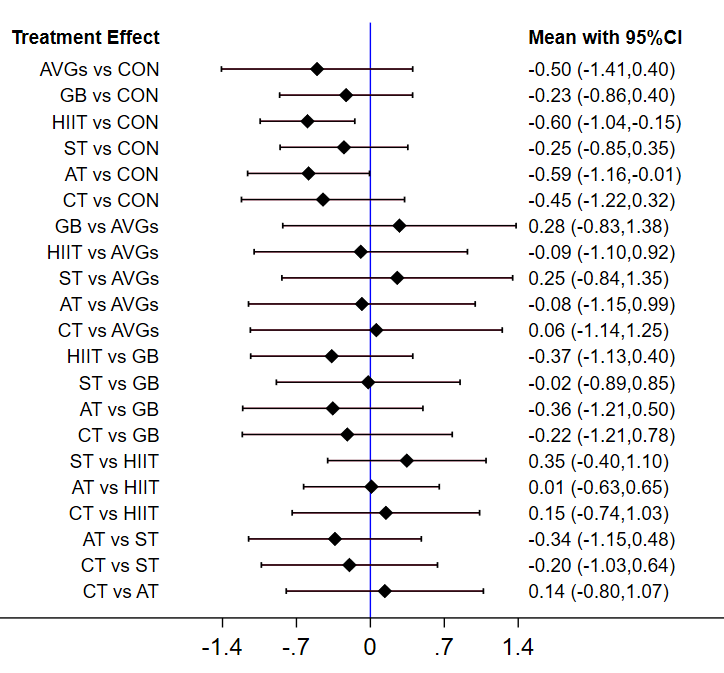


**Figure S11:** Forrest plot of eligible comparisons for body fat percent.

**
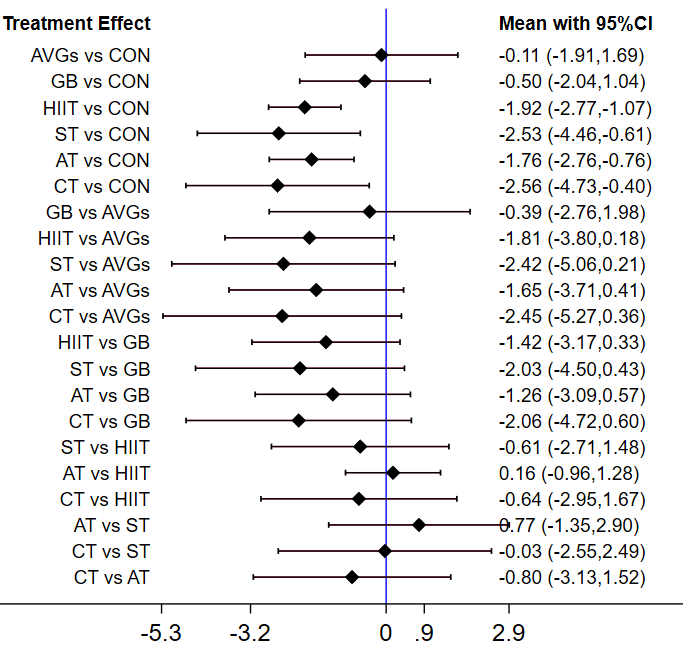
**

**Figure S12:** Forrest plot of eligible comparisons for waist circumstance.

**
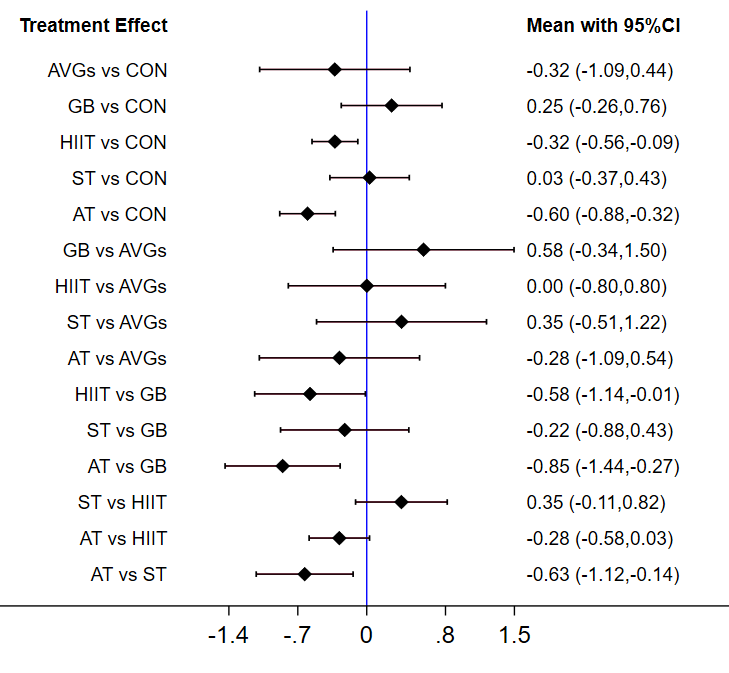
**

**Figure S13:** Forrest plot of eligible comparisons for standing long jump.


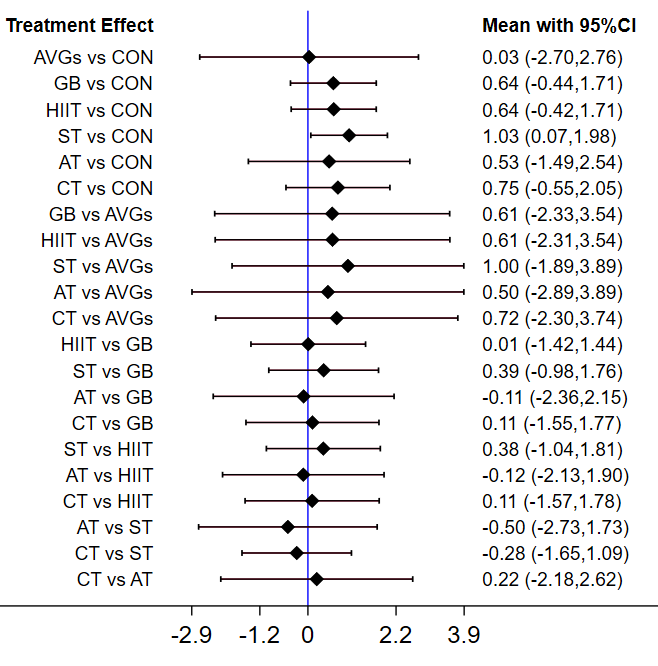


**Figure S14:** Forrest plot of eligible comparisons for countermovement jump

**
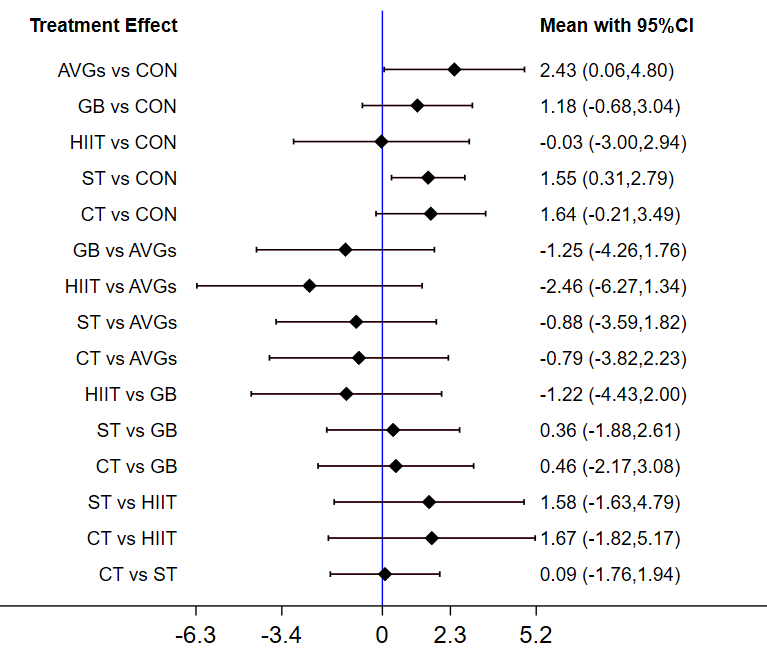
**

**Figure S15:** Forrest plot of eligible comparisons for push-ups


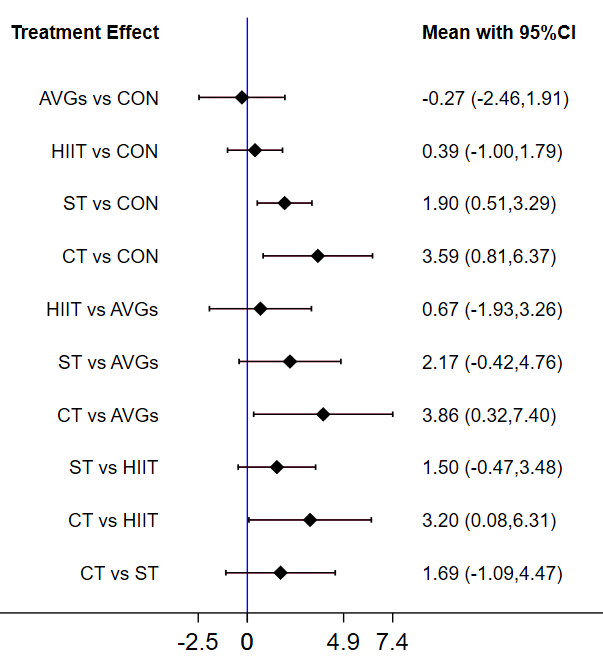


**Figure S16:** Forrest plot of eligible comparisons for 20-m sprint.

**
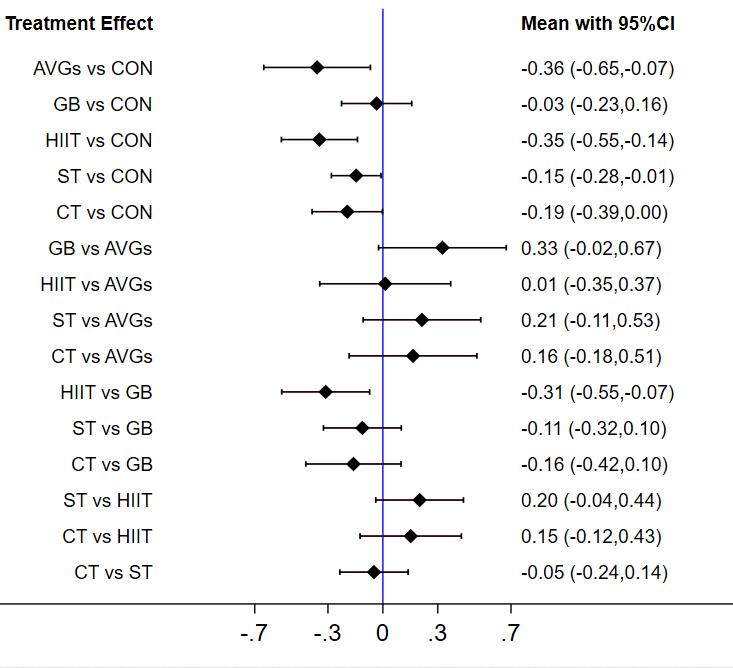
**

**Figure S17:** Forrest plot of eligible comparisons for shuttle running.


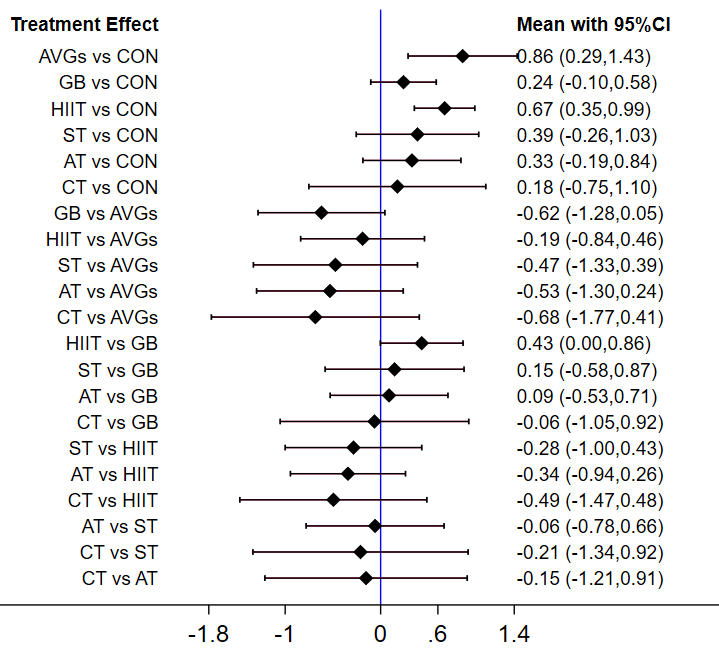


**Figure S18:** Forrest plot of eligible comparisons for VO_2max_.


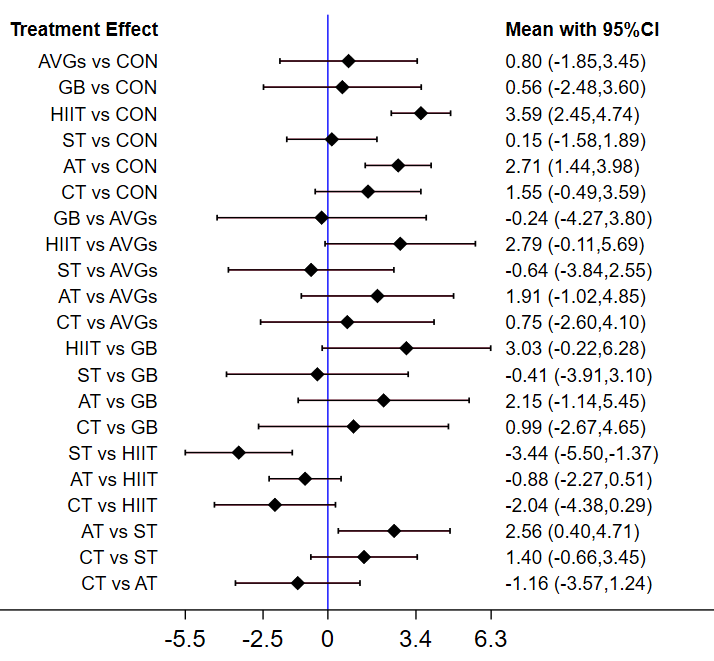


**Figure S19-S27:** Funnel plot graphics in this network meta-analysis.

**Figure S19:** Funnel plot graphics for BMI.


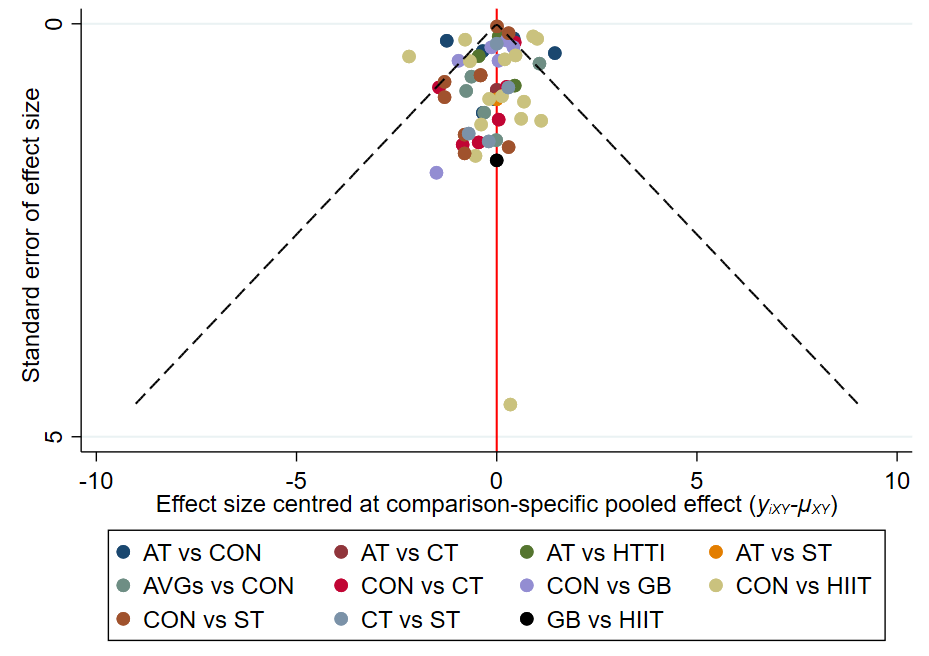


**Figure S20:** Funnel plot graphics for body fat percent.

**
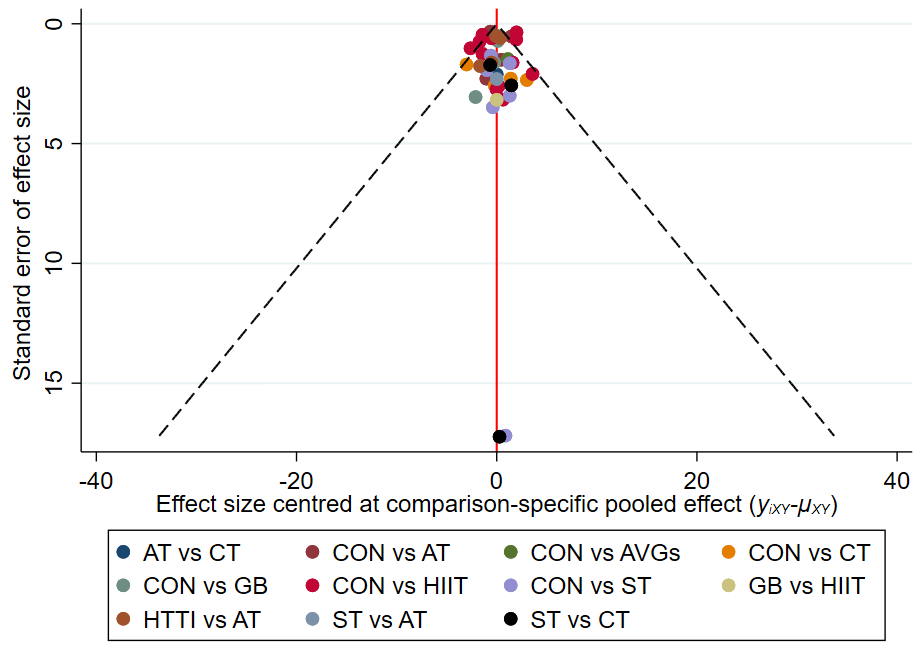
**

**Figure S21:** Funnel plot graphics for waist circumstance.

**
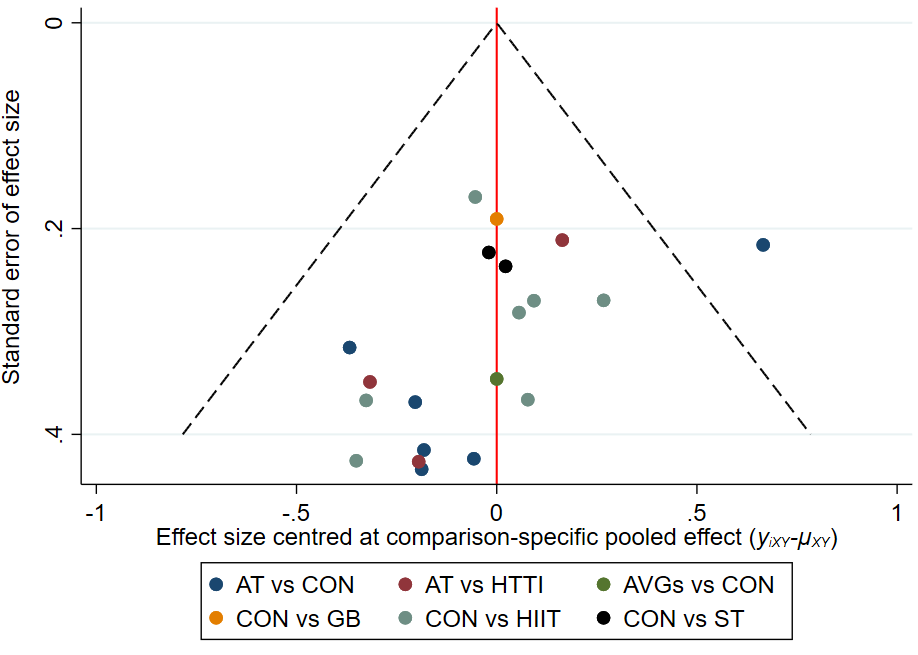
**

**Figure S22:** Funnel plot graphics for standing long jump.


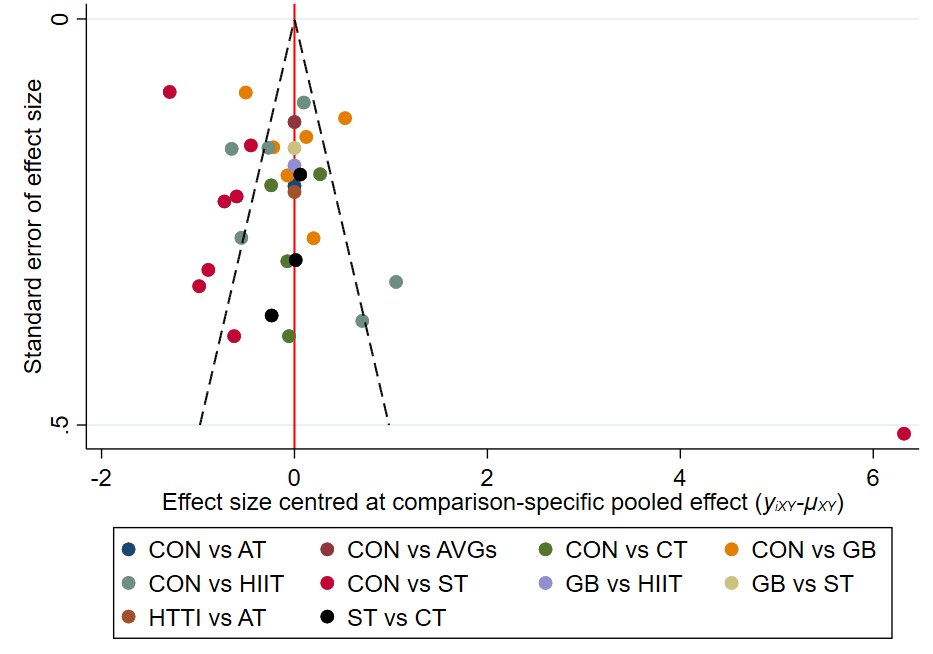


**Figure S23:** Funnel plot graphics for countermovement jump.

**
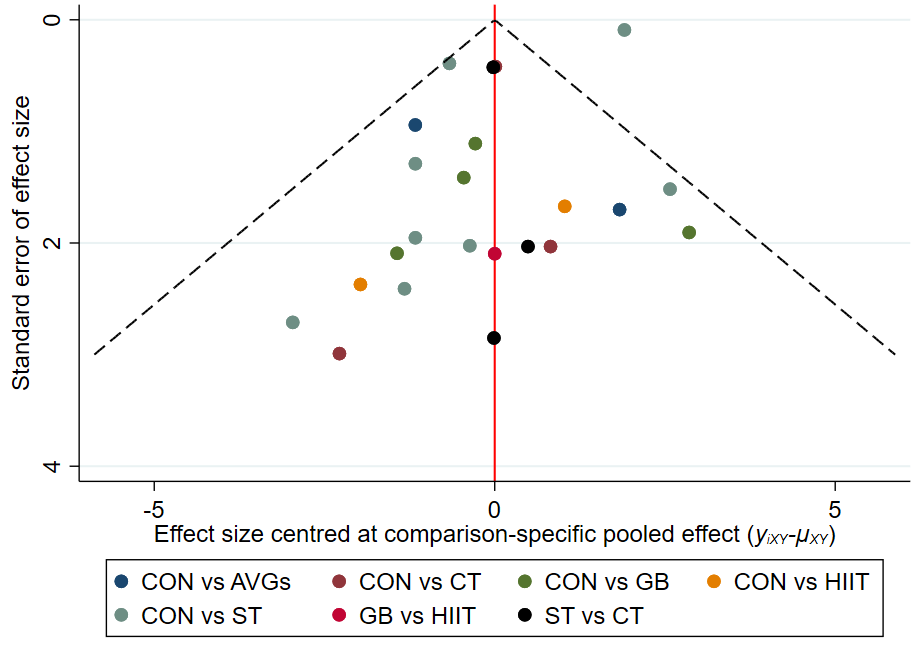
**

**Figure S24:** Funnel plot graphics for push-ups.


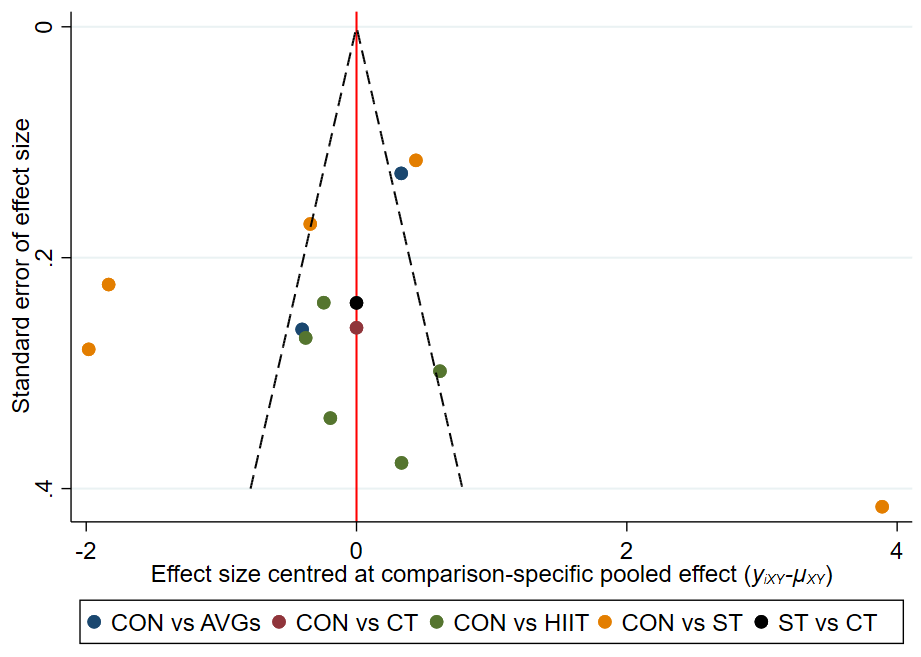


**Figure S25:** Funnel plot graphics for 20-m sprint.

**
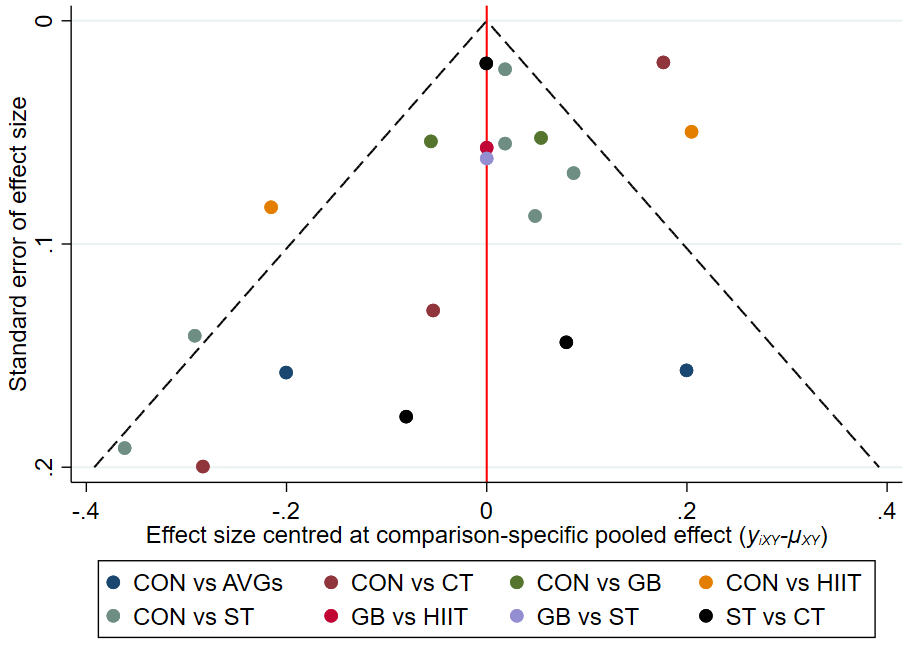
**

**Figure S26:** Funnel plot graphics for shuttle running.


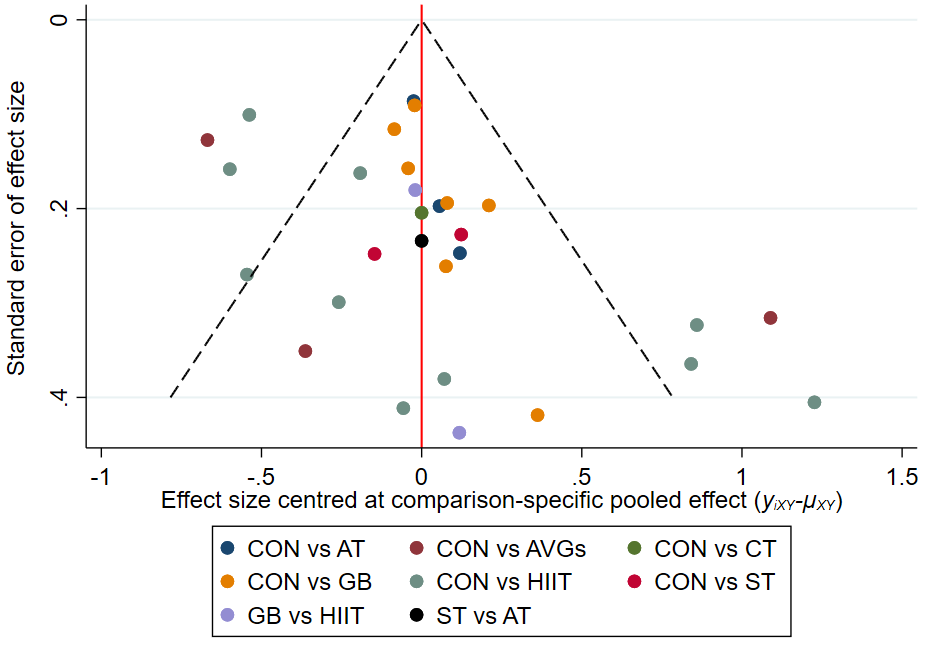


**Figure S27:** Funnel plot graphics for VO_2max_.


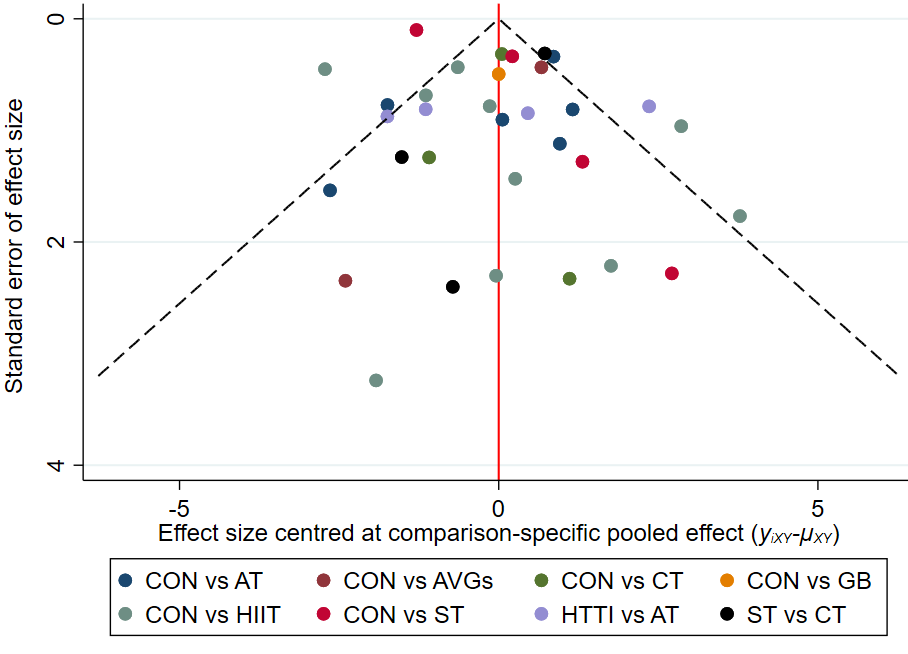


**Figure S28-S36:** SUCRA values for each intervention in this network meta-analysis

**Figure S28:** SUCRA values for BMI.


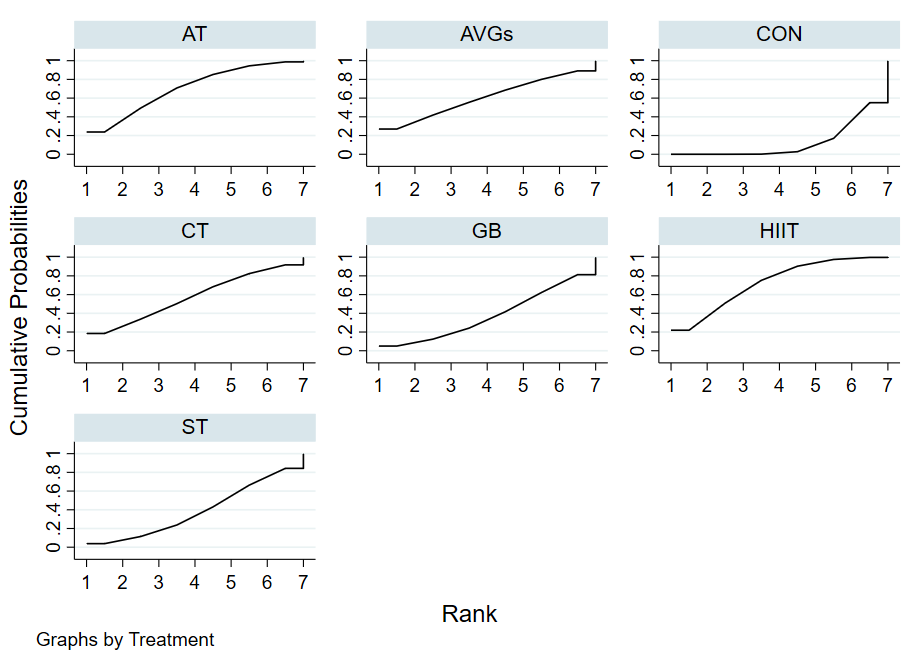


**Figure S29:** SUCRA values for body fat percent.

**
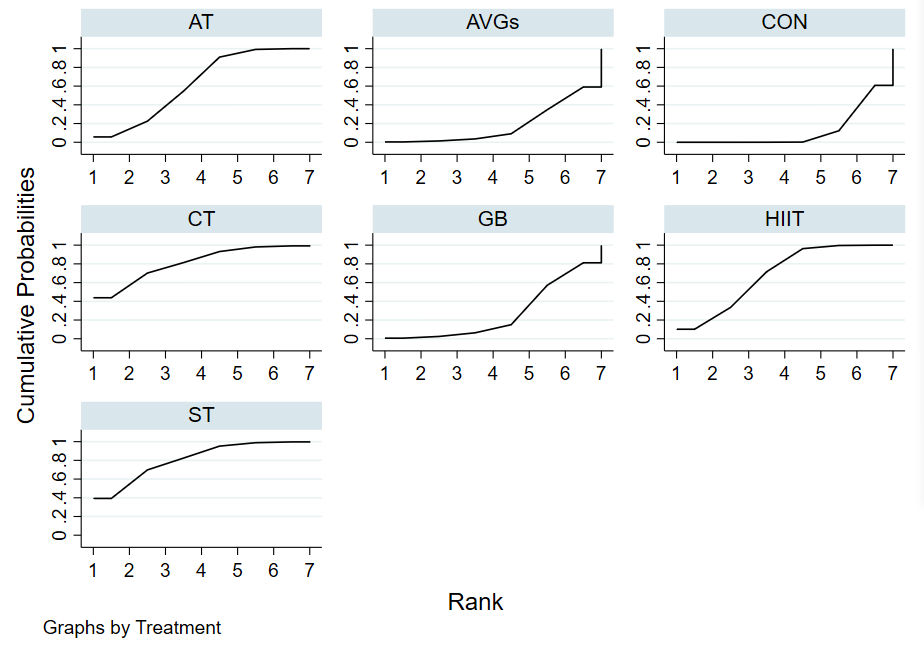
**

**Figure S30:** SUCRA values for waist circumstance.

**
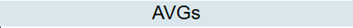

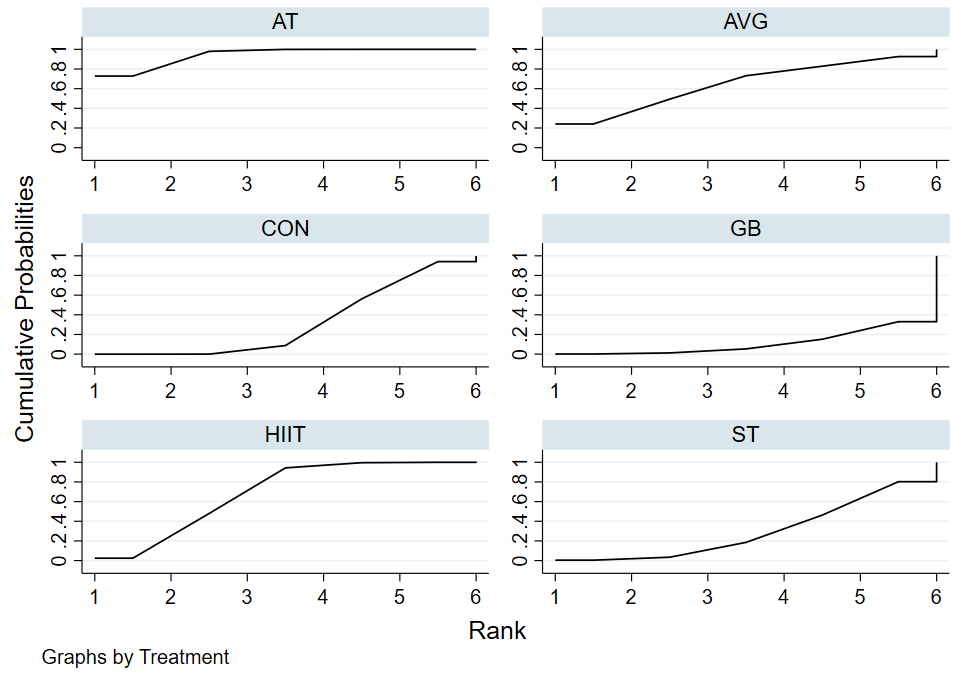
**

**Figure S31:** SUCRA values for standing long jump.

**
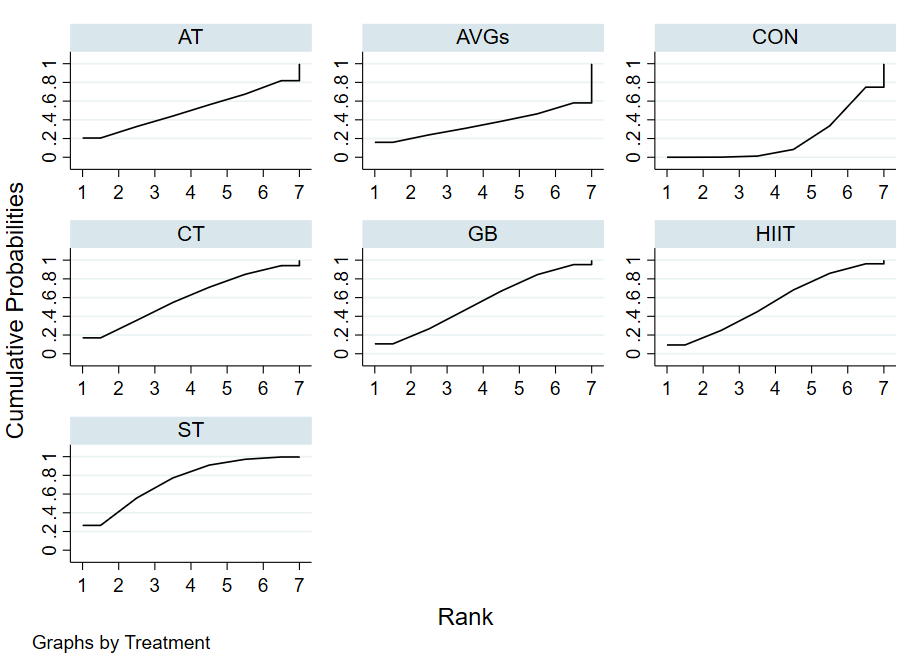
**

**Figure S32:** SUCRA values for countermovement jump.

**
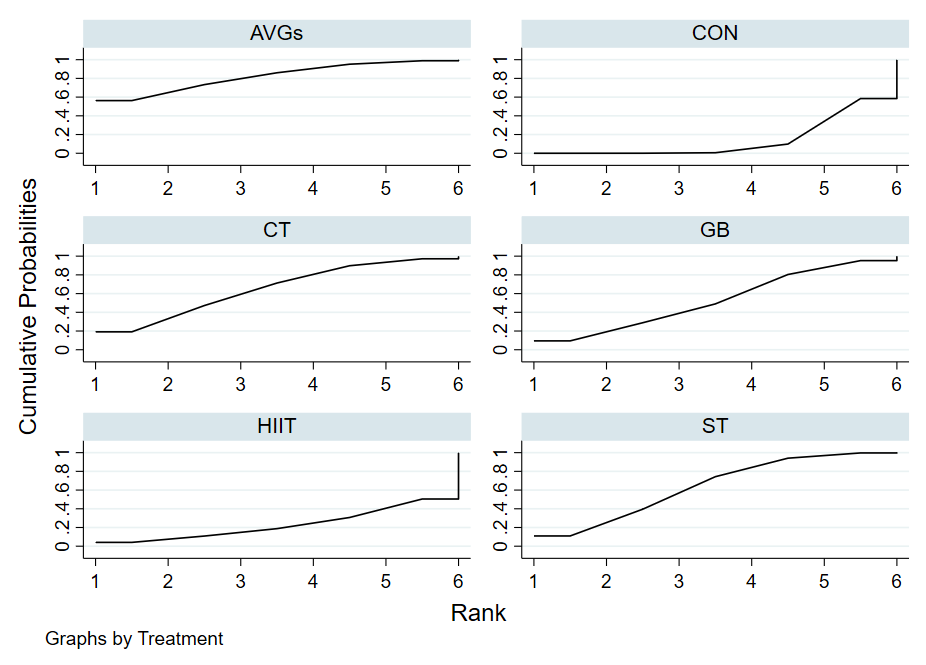
**

**Figure S33:** SUCRA values for push-ups.


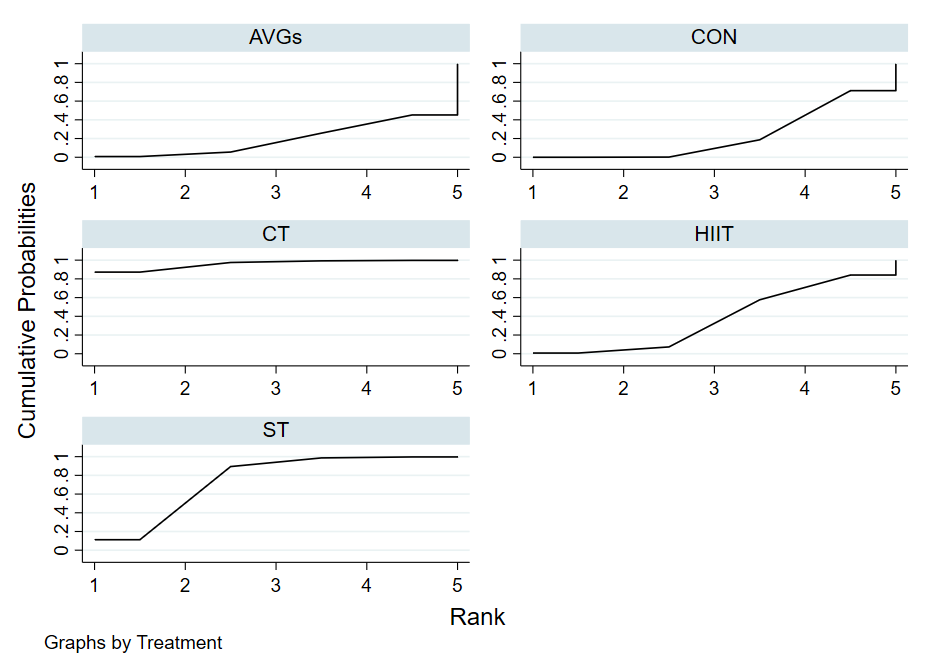


**Figure S34:** SUCRA values for 20-m sprint

**
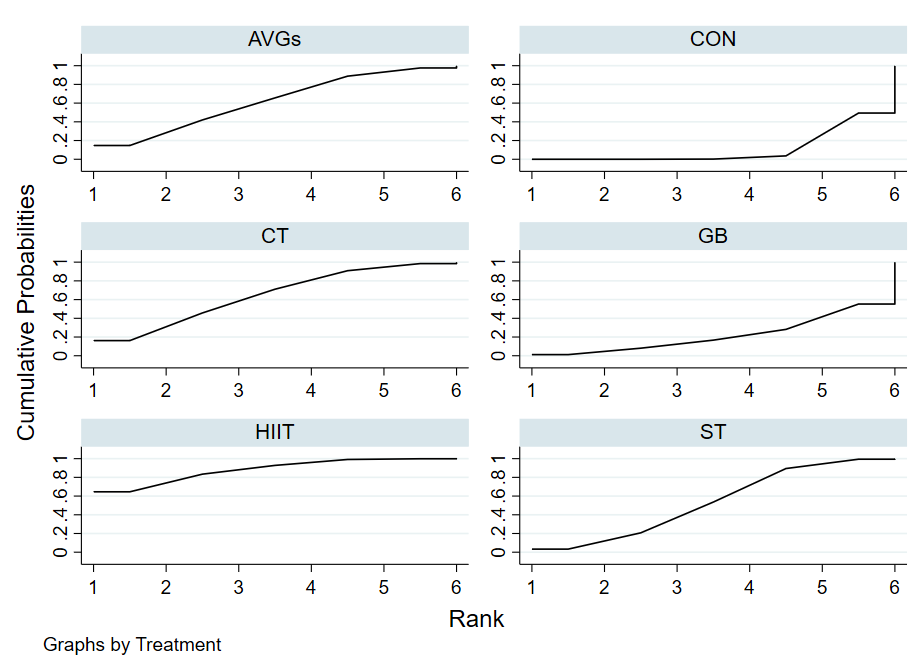
**

**Figure S35:** SUCRA values for shuttle running.


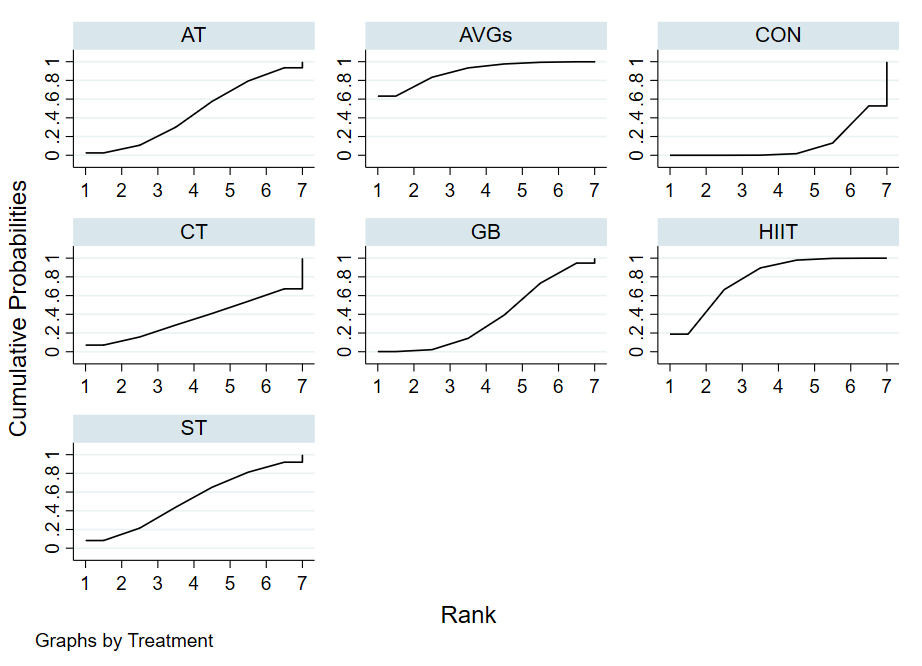


**Figure S36:** SUCRA values for VO_2max_.


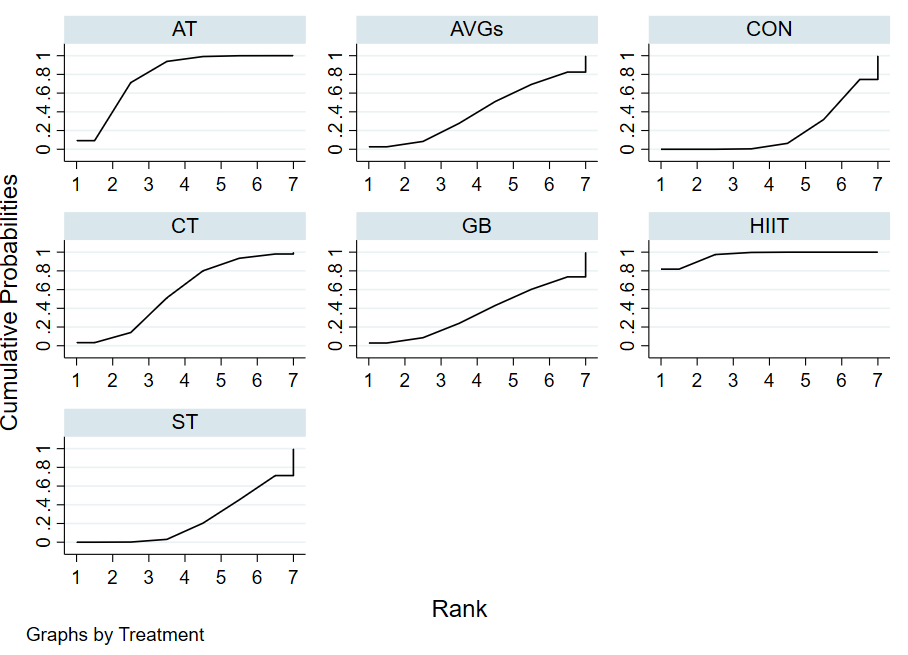


**Figure S37:** Cochrane Handbook Formulas


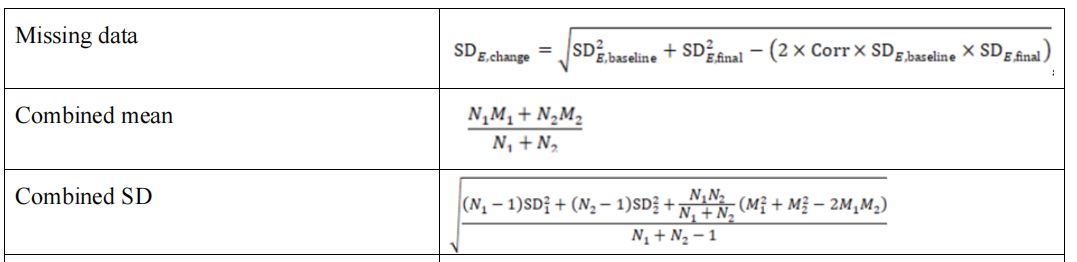


**Table S1:** Search strategy

|  | Search terms |
| --- | --- |
| 1. | child* [Title/Abstract] |
| 2. | youth* [Title/Abstract] |
| 3. | adolescents* [Title/Abstract] |
| 4. | pediatric* [Title/Abstract] |
| 5. | teen* [Title/Abstract] |
| 6. | Teenager* [Title/Abstract] |
| 7. | girl* [Title/Abstract] |
| 8. | boy* [Title/Abstract] |
| 9. | pupil* [Title/Abstract] |
| 10. | student* [Title/Abstract] |
| 11. | 1 OR 2 OR 3 OR 4 OR 5 OR 6 OR 7 OR 8 OR 9 OR 10 |
| 12. | school* [Title/Abstract] |
| 13. | Physical education* [Title/Abstract] |
| 14. | After school [Title/Abstract] |
| 15. | Before school [Title/Abstract] |
| 16. | 12 OR 13 OR 14 OR 15 |
| 17 | high-intensity interval training [Title/Abstract] |
| 18 | high-intensity interval exercise [Title/Abstract] |
| 19. | high-intensity training [Title/Abstract] |
| 20. | high-intensity exercise [Title/Abstract] |
| 21. | high-intensity intermittent training [Title/Abstract] |
| 22. | high-intensity intermittent exercise [Title/Abstract] |
| 23. | Intense intermittent training [Title/Abstract] |
| 24. | Intense intermittent exercise [Title/Abstract] |
| 25. | Intense interval training [Title/Abstract] |
| 26. | Intense interval exercise [Title/Abstract] |
| 27. | Sprint interval training [Title/Abstract] |
| 28. | 17 OR 18 OR 19 OR 20 OR 21 OR 22 OR 23 OR 24 OR 25 OR 26 OR 27 |
| 29. | active video gam* [Title/Abstract] |
| 30. | exergam* [Title/Abstract] |
| 31. | wii* [Title/Abstract] |
| 32. | Nintendo* [Title/Abstract] |
| 33. | xbox* [Title/Abstract] |
| 34. | Playstation* [Title/Abstract] |
| 35. | Kinect [Title/Abstract] |
| 36. | active videogam* [Title/Abstract] |
| 37. | interactive video gam* [Title/Abstract] |
| 38. | 29 OR 30 OR 31 OR 32 OR 33 OR 34 OR 35 OR 36 OR 37 |
| 39. | recreational football [Title/Abstract] |
| 40. | recreational soccer [Title/Abstract] |
| 41. | recreational basketball [Title/Abstract] |
| 42. | recreational handball [Title/Abstract] |
| 43. | recreational volleyball [Title/Abstract] |
| 44. | small-sided games [Title/Abstract] |
| 45. | 39 OR 40 OR 41 OR 42 OR 43 OR 44 |
| 46. | moderate-intensity continuous training [Title/Abstract] |
| 47. | moderate-intensity continuous exercise [Title/Abstract] |
| 48. | endurance training [Title/Abstract] |
| 49. | endurance exercise [Title/Abstract] |
| 50. | aerobic training [Title/Abstract] |
| 51. | aerobic exercise [Title/Abstract] |
| 52. | 46 OR 47 OR 48 OR 49 OR 50 OR 51 |
| 53. | strength training [Title/Abstract] |
| 54. | strength training [Title/Abstract] |
| 55. | resistance training [Title/Abstract] |
| 56. | resistance exercise [Title/Abstract] |
| 57. | weight training [Title/Abstract] |
| 58. | weight exercise [Title/Abstract] |
| 59. | 53 OR 54 OR 55 OR 56 OR 57 OR 58 |
| 60. | concurrent training [Title/Abstract] |
| 61. | concurrent exercise [Title/Abstract] |
| 62. | combined training [Title/Abstract] |
| 63. | combined exercise [Title/Abstract] |
| 64. | aerobic and resistance training [Title/Abstract] |
| 65. | aerobic and resistance exercise [Title/Abstract] |
| 66. | aerobic and strength training [Title/Abstract] |
| 67. | aerobic and strength exercise [Title/Abstract] |
| 68. | endurance and resistance training [Title/Abstract] |
| 69. | endurance and resistance exercise [Title/Abstract] |
| 70. | endurance and strength training [Title/Abstract] |
| 71. | endurance and strength exercise [Title/Abstract] |
| 72. | 60 OR 61 OR 62 OR 63 OR 64 OR 65 OR 66 OR 67 OR 68 OR 69 OR 70 OR 71 |
| 73. | Physical fitness [Title/Abstract] |
| 74. | Health-related fitness [Title/Abstract] |
| 75. | Body composition [Title/Abstract] |
| 76. | Muscular fitness [Title/Abstract] |
| 77. | Cardiovascular fitness [Title/Abstract] |
| 78. | Cardiorespiratory fitness [Title/Abstract] |
| 79. | Cardiometabolic risk [Title/Abstract] |
| 80. | Body fat [Title/Abstract] |
| 81. | Aerobic fitness [Title/Abstract] |
| 82. | 73 OR 74 OR 75 OR 76 OR 77 OR 78 OR 79 OR 80 OR 81 |
| 83. | 28 OR 38 OR 45 OR 52 OR 59 OR 72 |
| 84. | 11 AND 16 AND 83 AND 82 |

**Table S2:** Risk of bias results

| Study | Randomization | Allocation concealed | Blinding of participant | Blinding of research personnel | Incomplete outcome data | Selective reporting | Other risk of bias | Overall category |
| --- | --- | --- | --- | --- | --- | --- | --- | --- |
| Cohen et al.,2021 | High | High | High | High | Low | Low | Low | Moderate |
| Yoshimoto et al.2016 | High | High | High | High | Low | Low | Unclear | Moderate |
| Sun et al.2011 | High | High | High | High | Low | Low | Low | Moderate |
| Petrusic et al.,2022 | High | High | High | High | Low | Unclear | Low | Moderate |
| Trajkovic et al.,2021 | High | High | High | High | Low | Unclear | Low | Moderate |
| Trajkovic et al.,2020a | High | High | High | High | Low | Low | Unclear | Moderate |
| Trajkovic et al.,2020b | High | High | High | High | Low | Unclear | Unclear | Moderate |
| Lau et al.,2016 | High | Low | High | High | Low | Unclear | Unclear | Low |
| Liang et al.,2019 | High | High | High | High | Low | Low | High | High |
| Ketelhut et al.,2022 | Low | Unclear | High | High | Low | Unclear | Unclear | Low |
| Comeras-Chueca et al.,2022a | Low | Unclear | High | High | Low | Unclear | Unclear | Low |
| Ye et al.,2018 | High | High | High | High | Low | Unclear | High | High |
| Comeras-Chueca et al.,2022b | Low | Unclear | High | High | Low | Unclear | Unclear | Low |
| Chen et al.,2017 | High | High | High | High | Low | Unclear | High | High |
| Lau et al.,2020 | High | High | High | Low | Low | Low | Low | Moderate |
| Camacho-Cardenosa et al.,2016 | High | High | High | High | Low | Unclear | Unclear | Moderate |
| Cao et al.,2022 | Low | High | High | High | Low | Low | Unclear | Moderate |
| Cvetkovic et al.,2018 | Low | High | High | High | Low | Low | Unclear | Moderate |
| Delgado-Floody et al.,2018 | High | High | High | High | High | Unclear | High | High |
| Martin et al.,2015 | High | High | High | High | Low | Unclear | Unclear | Moderate |
| Martin-Smith et al.,2019 | High | High | High | High | Low | Unclear | Unclear | Moderate |
| Martinez-Vizcaino et al.,2022 | High | High | High | High | High | Low | Low | High |
| Meng et al.,2022 | Low | Low | High | High | Low | Low | Low | Low |
| Alonso-Fernández et al.,2019 | High | High | High | High | Low | Low | Unclear | Moderate |
| Bogataj et al.,2021 | High | High | High | High | Low | Low | Low | Moderate |
| Costigan et al.,2015 | High | High | High | High | Low | Low | Low | Moderate |
| Larsen et al.,2017 | Low | Unclear | High | Low | Low | Low | Low | Low |
| Alves et al.,2016 | High | High | High | High | Low | Low | Unclear | Moderate |
| Santos et al., 2011 | High | High | High | High | Low | Low | High | High |
| Dorgo et al.,2009 | High | High | High | High | High | Low | Unclear | High |
| Santos et al., 2012 | High | High | High | High | Low | Low | Unclear | Moderate |
| Kennedy et al.,2018 | High | High | High | High | High | Low | Low | High |
| Eather et al.,2016 | Low | Unclear | High | High | Low | Low | Unclear | Low |
| Zhao et al.,2022 | High | High | High | High | Low | Low | Low | Moderate |
| Muehlbauer et al.,2012 | High | High | High | High | Low | Unclear | Unclear | Moderate |
| Martínez et al.,2016 | High | High | High | High | Low | Unclear | Unclear | Moderate |
| van der Fels et al.,2020 | High | High | High | High | High | Low | Low | High |
| Latorre-Roman et al.,2018 | Low | High | High | High | Low | Unclear | Low | Low |
| Engel et al.,2019 | High | High | High | High | Low | Unclear | Unclear | Moderate |
| Alves et al.,2017 | Low | High | High | High | Low | Low | Low | Low |
| Krustrup et al.,2014 | High | High | High | Unclear | Low | Low | Low | Low |
| Skoradal et al.,2018 | High | High | High | High | Low | Low | Low | Moderate |
| Larsen et al.,2018 | Low | High | High | High | Low | Low | Low | Low |
| Ryom et al.,2022 | High | High | High | High | High | Unclear | Unclear | High |
| Cohen et al.,2022 | High | High | High | High | Low | Unclear | Low | Moderate |
| Lubans et al.,2010 | Low | High | High | High | Low | Unclear | Low | Low |
| Lambrick et al.,2016 | Low | High | High | High | Low | Unclear | Unclear | Low |
| Tan et al.,2017 | High | High | High | High | Low | Unclear | Unclear | Moderate |
| Walther et al.,2009 | High | High | High | Low | Low | Low | Low | Low |
| Baquet et al.,2010 | High | High | High | High | Low | Unclear | Unclear | Moderate |
| Baquet et al., 2004 | High | High | High | High | Low | Unclear | Unclear | Moderate |
| Gamelin et al.,2009 | High | High | High | High | Low | Unclear | Unclear | Moderate |
| Granacher et al.,2011 | High | High | High | High | Low | Unclear | Low | Moderate |
| Leahy et al.,2019 | High | High | High | Low | Low | Low | Unclear | Low |
| McNarry et al.,2020 | Low | High | High | High | Low | Unclear | Unclear | Low |
| Racil et al.,2013 | High | High | High | High | Low | Unclear | Unclear | Moderate |
| Racil et al.,2016 | High | High | High | High | Low | Unclear | Unclear | Moderate |
| Winwood et al.,2019 | High | High | High | High | Low | Unclear | Unclear | Moderate |
| Harris et al., 2021 | Low | High | High | High | Low | Unclear | Low | Low |
| Juric et al.,2023 | Low | High | High | High | High | Low | Low | Moderate |
| Marta et al.,2019 | Low | High | High | High | Low | Unclear | Low | Low |
| Robinson et al .,2022 | High | High | High | High | Low | Low | Unclear | Moderate |
| Song et al.,2012 | High | High | High | High | Low | Unclear | Unclear | Moderate |
| Cataldi et al.,2021 | Low | High | High | High | Low | Unclear | Unclear | Moderate |
| Velez et al.,2010 | High | High | High | High | Low | Unclear | Unclear | Moderate |
| Wong et al.,2008 | High | High | High | High | Low | Unclear | Low | Moderate |

**Table S3:** Summary of included studies

| Study | Duration (wks) | Exercise category | Sample size M/F | Mean age (SD) or age range | Description of exercise intervention  (frequency, intensity, time and type) | Outcome measures reported |
| --- | --- | --- | --- | --- | --- | --- |
| Choen et al.,2021 | 16 | ST | 22/18 | 13-17 | 2d/wk; progressive intensity started from 16–20RM; 50min; Multigym (machine) exercises, free-weights; bodyweight and Functional ST | 20-m sprint |
|  |  | AT | 19/21 |  | 2d/wk; progressive intensity started from 65–75% HR_max_; 50min; multi-directional jogging/running |  |
|  |  | CON | 15/15 |  | regular PE/PA |  |
| Yoshimoto et al.,2016 | 8 | ST | 0/27 | 13.8 ± 0.6 | 5-6d/wk；body mass-based squat exercise training (100 reps/day, 45 sessions) | BMI, BF% |
|  |  | CON | 0/20 |  | regular PE/PA |  |
| Sun et al.,2011 | 10 | AT | 18/7 | 13.6 ± 0.7 | 4d/wk；60min；40%–60%VO_2max_；jogging, running, jumping rope, and group activities | BMI, WC, VO_2max_ |
|  |  | CON | 7/10 |  | regular PE/PA |  |
| Petrusic et al.,2022 | 12 | GB | 0/3 | 13.3 ± 0.3 | 2d/wk；40min；small-sided ball games | SLJ, CMJ, SR |
|  |  | CON | 0/29 | 13.2 ± 0.4 | regular PE/PA |  |
| Trajkovic et al.,2021 | 12 | GB | 0/20 | 15.6 ± 0.5 | 2d/wk; 57min; volleyball games | BMI, BF% |
|  |  | CON | 0/22 | 15.5 ± 0.7 | regular PE/PA |  |
| Trajkovic et al.,2020a | 32 | GB | 38/17 | 14~16 | 2d/wk; 45min; volleyball games | CMJ, SR |
|  |  | CON | 35/19 |  | regular PE/PA |  |
| Trajkovic et al.,2020b | 32 | GB | 40/14 | 15.7 ± 0.6 | 2d/wk; 45min; football games | CMJ, SR |
|  |  | CON | 35/16 | 15.8 ± 0.5 | regular PE/PA |  |
| Lau et al.,2016 | 12 | AVGs | 28/13 | 8~11 | 2d/wk; 60min; team games in Xbox360 and Kinect | BMI, VO2_max_ |
|  |  | CON | 27/12 |  | regular PE/PA |  |
| Liang et al.,2019 | 8 | AVGs | 24/6 | 10.5 ± 0.7 | 2d/wk; 60min; Kinect “Sports”； | BF% |
|  |  | CON | 30/27 | 10.4 ± 0.8 | regular PE/PA |  |
| Ketelhut et al.,2022 | 12 | AVGs | 8/10 | 10.4 ± 0.8 | 2d/wk; 15-20min; 90% HR_max_; Sphery Racer； | BMI, WC, CMJ, 20-m SRT |
|  |  | CON | 9/7 |  | regular PE/PA |  |
| Comeras-Chueca et al.,2022a | 20 | AVGs | 12/9 | 10.07 ± 0.84 | 3d/wk；90%HR_max_；60min; AVGs included Xbox360 with Kinect using “Kinect Adventures” and “KinectSports”, the Nintendo Wii using “Wii Sports” “Just Dance”and “Mario and Sonic at the Olympic Games” | BMI, CMJ, 20-m sprint |
|  |  | CON | 4/4 |  | regular PE/PA |  |
| Ye et al.,2018 | 36 | AVGs | 65/70 | 8.27 ± 0.70 | 3d/wk; 25min; Kinect Ultimate Sports, Just Dance, Wii Sports, and Wii Fit； | SLJ, push-ups, SR |
|  |  | CON | 56/59 |  | regular PE/PA |  |
| Comeras-Chueca et al.,2022b | 20 | AVGs | 11/9 | 10.07 ± 0.84 | 3d/wk; 90%HR_max_; 60min; AVGs included Xbox360 with Kinect using “Kinect Adventures” and “KinectSports”, the Nintendo Wii using “Wii Sports” “Just Dance”and “Mario and Sonic at the Olympic Games” | BMI, BF%, VO_2max_ |
|  |  | CON | 4/4 |  | regular PE/PA |  |
| Chen et al.,2017 | 16 | AVGs | 15/21 | 8~11 | 3d/wk; 40min; Zumba Kids and Just Dance Kids 2014 | push-ups, SR |
|  |  | CON | 10/19 |  | regular PE/PA |  |
| Lau et al.,2020 | 12 | AVGs | 92/33 | 8~18 | 2d/wk; 30min; team games in Xbox360 and Kinect | BMI, BF% |
|  |  | CON | 54/24 |  | regular PE/PA |  |
| Camacho-Cardenosa et al.,2016 | 8 | HIIT | 10/8 | 11.06 ± 0.24 | 3d/wk; 50min; running-based HIIT； | BF% |
|  |  | CON | 9/8 | 11.29 ± 0.47 | regular PE/PA |  |
| Cao et al.,2022 | 12 | HIIT | 10/10 | 11.0 ± 0.6 | 3d/wk; 35min; running-based HIIT； | BMI, BF%, VO_2max_, 20-m SRT |
|  |  | CON | 10/10 |  | regular PE/PA |  |
| Cvetkovic et al.,2018 | 12 | HIIT | 11/0 | 11~13 | 3d/wk; 60min; running-based HIIT | BMI, BF%, CMJ, SR |
|  |  | GB | 10/0 |  | 3d/wk; 60min; football games |  |
|  |  | CON | 14/0 |  | regular PE/PA |  |
| Delgado-Floody et al.,2018 | 28 | HIIT | 70/81 | 8.39 ± 1.15 | 2d/wk; 60min; running, jumping, throwing | BMI, BF%, WC |
|  |  | CON | 11/18 |  | regular PE/PA |  |
| Martin et al.,2015 | 7 | HIIT | 13/7 | 16.9 ± 0.4 | 3d/wk; 15min; running-based HIIT | BMI, VO_2max_, 20-m sprint |
|  |  | CON | 18/5 | 16.8 ± 0.5 | regular PE/PA |  |
| Martin-Smith et al.,2019 | 4 | HIIT | 13/9 | 17.0 ± 0.3 | 3d/wk;10min; running-based HIIT | WC, VO_2max_ |
|  |  | CON | 19/11 | 16.8 ± 0.5 | regular PE/PA |  |
| Martinez-Vizcaino et al.,2022 | 32 | HIIT | 120/128 | 9~11 | 4d/wk; 60min; HIIT during extracurricular hours | BMI, BF%, WC, SLJ, VO_2max_, 20-m SRT |
|  |  | CON | 113/126 |  | regular PE/PA |  |
| Meng et al.,2022 | 12 | HIIT | 12/0 | 11.2 ± 0.7 | 3d/wk; 15min; >90% maximal aerobic speed; running-based HIIT | BMI, BF%, WC, VO_2max_ |
|  |  | AT | 11/0 |  | 3d/wk; 60~70%; 30min; running |  |
|  |  | CON | 13/0 |  | regular PE/PA |  |
| Alonso-Fernández et al.,2019 | 6 | HIIT | 15/6 | 15~16 | 2d/wk; 6min; squat jump, push up, high skipping, burpees, isometric front plank, multi jumps on the bench, mountain climbers: skipping in hand plank, lateral sprints of 5 meters； | BMI, BF%, VO_2max_ |
|  |  | CON | 8/5 |  | regular PE/PA |  |
| Bogataj et al.,2021 | 8 | HIIT | 0/24 | 15.5 ± 0.7 | 3d/wk;15min; push-up on knees, squats, burpees, crunches, step-up, triceps dips, hop jumps, chest medicine ball throws against the wall, skipping rope, and plank | BMI, BF%, CMJ, SR |
|  |  | CON | 0/24 | 15.7 ± 0.6 | regular PE/PA |  |
| Costigan et al.,2015 | 8 | HIIT | 31/12 | 15.8 ± 0.6 | 3d/wk; 10min; shuttle runs, jumping jacks, skipping | BMI, WC, SLJ, push-ups, SR |
|  |  | CON | 14/8 |  | regular PE/PA |  |
| Larsen et al.,2017 | 40 | HIIT | 31/30 | 8~10 | 5d/wk; 12min; running-based HIIT； | SLJ, SR, 20-m sprint |
|  |  | GB | 31/31 |  | 5d/wk; 12min; small-sided football plus other ball games； |  |
|  |  | CON | 58/58 |  | regular PE/PA |  |
| Alves et al.,2016 | 8 | ST | 19/22 | 10~11 | 2d/wk；45min; 1 and 3kg ball throw; jump;20-m sprint running | SLJ, VO_2max_, CMJ, 20-m sprint |
|  |  | CT | 42/41 |  | 2d/wk；60min; concurrent training in same or different sessions |  |
|  |  | CON | 21/23 |  | regular PE/PA |  |
| Santos et al., 2011 | 8 | ST | 0/21 | 13.5 ± 1.03 | 2d/wk；approximately 30min; chest 1 and 3kg medicine ball throw, overhead 1 and 3kg medicine ball throw, jump, sprinting | BMI, BF%, SLJ, VO_2max_, CMJ, 20-m sprint |
|  |  | CT | 0/25 |  | 2d/wk；approximately 35min; approximately 30min; chest 1 and 3kg medicine ball throw, overhead 1 and 3kg medicine ball throw, jump, sprinting plus 20m shuttle run |  |
|  |  | CON | 0/21 |  | regular PE/PA |  |
| Dorgo et al.,2009 | 18 | ST | 37/30 | 15~16 | 3d/wk;80min; manual resistance training； | BMI, Push-up |
|  |  | CT | 41/33 |  | 3d/wk;80min; manual resistance training plus cardiovascular endurance training with >60% HR_max_ (walking, jogging, step aerobics and aerobic kick-boxing) |  |
|  |  | CON | 127/105 |  | regular PE/PA |  |
| Santos et al., 2012 | 8 | ST | 15/0 | 13.3 ± 1.04 | 2d/wk；approximately 30min; chest 1 and 3kg medicine ball throw, overhead 1 and 3kg medicine ball throw, jump, sprinting | BMI, BF%, SLJ, VO_2max_, CMJ, 20-m sprint |
|  |  | CT | 15/0 |  | 2d/wk；approximately 35min; chest 1 and 3kg medicine ball throw, overhead 1 and 3kg medicine ball throw, jump, sprinting plus 20m shuttle run |  |
|  |  | CON | 12/0 |  | regular PE/PA |  |
| Kennedy et al.,2018 | 24 | ST | 179/174 | 14.1 ± 0.5 | 90min/wk; structured strength training program | BMI, SLJ, VO_2max_, push-ups |
|  |  | CON | 124/130 |  | regular PE/PA |  |
| Eather et al.,2016 | 8 | ST | 24/31 | 15.4 ± 0.5 | 2d/wk; 60min; combinations of core strength exercises | BMI, WC, SLJ, push-ups, 20m-SRT |
|  |  | CON | 22/19 |  | regular PE/PA |  |
| Zhao et al.,2022 | 10 | ST | 70/0 | 13.46 ± 0.6 | 3d/wk; 20-25min; a circuit of 6–8 exercise stations | SLJ, CMJ, push-ups |
|  |  | CON | 71/0 |  | regular PE/PA |  |
| Muehlbauer et al.,2012 | 8 | ST | 6/8 | 16~17 | 2d/wk;90min; squats, leg-press, calf-raise, hip abduction/adduction, leg extension/ flexion | CMJ |
|  |  | CON | 7/7 |  | regular PE/PA |  |
| Martínez et al.,2016 | 12 | HIIT | 18/20 | 7~9 | 2d/wk; 40min; half-squats followed by sprints and a training circuit consisting of jumps, speed/agility tasks, carrying weights, pulling exercises | BMI, BF%, WC, SLJ, VO_2max_ |
|  |  | AT | 34/22 |  | 2d/wk; moderate intensity; 40min; aerobic exercises |  |
| van der Fels et al.,2020 | 14 | AT | 108/113 | 9.2 ± 0.7 | 4d/wk; 40min; highly repetitive and automated exercises，team exercises | 20m-SRT |
|  |  | CON | 219/211 |  | regular PE/PA |  |
| Latorre-Roman et al.,2018 | 10 | GB | 28/28 | 4.42 ± 0.53 | 3d/wk; 30min; aerobic games, or gross locomotor movement | BMI, WC, SLJ, 20-m sprint |
|  |  | CON | 32/33 | 4.43 ± 0.62 | regular PE/PA |  |
| Engel et al.,2019 | 4 | HIIT | 11/6 | 11.6 ± 0.2 | 4d/wk；>85% HR_max_；6.0 ± 1.5min; functional HIIT training； | SLJ, push-ups, 20-m sprint |
|  |  | CON | 11/7 |  | regular PE/PA |  |
| Alves et al.,2017 | 8 | ST | 0/22 | 10~11 | 2d/wk；approximately 40min; upper body and lower body strength training plus a speed drill； | BMI, BF% |
|  |  | CT | 0/81 |  | 2d/wk; approximately 45min; 20m shuttle run exercise combine with upper body and lower body strength exercises, as well as a speed drill； |  |
|  |  | CON | 0/23 |  | regular PE/PA |  |
| Krustrup et al.,2014 | 10 | GB | 21/30 | 9~10 | 3d/wk; 71 ± 6% HR_max_; 40min; football game | BMI |
|  |  | CON | 23/23 |  | regular PE/PA |  |
| Skoradal et al.,2018 | 11 | GB | 146/146 | 10~12 | 2d/wk; 45min; small-side ball game； | BMI, BF%, SLJ, SR |
|  |  | CON | 57/43 |  | regular PE/PA |  |
| Larsen et al.,2018 | 40 | GB | 46/50 | 8~10 | 3d/wk; 40min; ball games； | SLJ, 20-m sprint |
|  |  | ST | 43/40 |  | 3d/wk; 40min; traditional circuit training with strength exercises |  |
|  |  | CON | 55/61 |  | regular PE/PA |  |
| Ryom et al.,2022 | 11 | GB | 507/437 | 10~12 | 2d/wk; 45min; small-sided games, and health education； | SLJ, SR |
|  |  | CON | 89/89 |  | regular PE/PA |  |
| Cohen et al.,2022 | 22 | CT | 0/41 | 13~17 | 2d/wk; 60min; combination of aerobic training strength training phase | BMI, SLJ, 20-m sprint |
|  |  | CON | 0/58 |  | regular PE/PA |  |
| Lubans et al.,2010 | 8 | ST | 14/16 | 15 ± 0.7 | 2d/wk; 40-50min; squat, lunge, calf raise, bent over row, bench press, front raise, biceps curl, triceps extension, crunch and Russian twist | BMI, BF%, WC |
|  |  | CON | 42/36 |  | regular PE/PA |  |
| Lambrick et al.,2016 | 6 | HIIT | 18/10 | 8~10 | 2d/wk; high intensity; 40min; multiple physical activities | BMI, BF%, WC, VO_2max_ |
|  |  | CON | 14/13 |  | regular PE/PA |  |
| Tan et al.,2017 | 10 | AT | 27/25 | 5 | 5d/wk; 50% heart rate reserve; 60min; quick walking, slow running, jumping, rope skipping, semi-squatting, and slow crawling | BMI, BF%, WC, SLJ, 20m- sprint |
|  |  | CON | 26/26 |  | regular PE/PA |  |
| Walther et al.,2009 | 50 | AT | 58/51 | 11.1 ± 0.7 | 2d/wk; 45min; endurance training | VO_2max_ |
|  |  | CON | 42/31 |  | regular PE/PA |  |
| Baquet et al.,2010 | 7 | HIIT | 11/11 | 8-11 | 3d/wk; 18-39min; running-based HIIT； | VO_2max_ |
|  |  | AT | 12/10 |  | 3d/wk; 18-39min; light intensity; running |  |
|  |  | CON | 9/10 |  | regular PE/PA |  |
| Baquet et al., 2004 | 7 | HIIT | 21/26 | 9.7 ± 0.8 | 2d/wk; 30min; short high-intensity; intermittent-running aerobic exercises | BF% |
|  |  | CON | 25/28 |  | regular PE/PA |  |
| Gamelin et al.,2009 | 7 | HIIT | 15/11 | 9.6 ± 1.2 | 3d/wk; 30min; short intermittent runs with exercise/recovery sequences | VO_2max_ |
|  |  | CON | 14/12 |  | regular PE/PA |  |
| Granacher et al.,2011 | 10 | ST | 8/9 | 8.6 ± 0.1 | 2d/wk；70-80 % 1RM; 90min;leg-press, knee extension / flexion，seated calf-raise, weight-machine for hip abduction/ adduction, core exercises; | CMJ |
|  |  | CON | 10/5 | 8.5 ± 0.1 | regular PE/PA |  |
| Leahy et al.,2019 | 14 | HIIT | 21/17 | 16~18 | 3d/wk; 12-20min; multiple HIIT | BMI, SLJ, 20-m sprint |
|  |  | CON | 16/14 |  | regular PE/PA |  |
| McNarry et al.,2020 | 24 | HIIT | 9/8 | 13.6 ± 0.9 | 3d/wk; 30min; a mixture of circuits and games-based high intense activities | BMI |
|  |  | CON | 9/7 |  | regular PE/PA |  |
| Racil et al.,2016 | 12 | HIIT | 0/11 | 15.9 ± 0.3 | 3d/wk; 20min; running-based HIIT | BF%, WC, VO_2max_ |
|  |  | AT | 0/11 |  | 3d/wk; 20min; light intense running； |  |
|  |  | CON | 0/12 |  | regular PE/PA |  |
| Racil et al.,2016 | 12 | HIIT | 0/17 | 14.2 ± 1.2 | 3d/wk; 100%maximal aerobic speed; 50-60min; HIIT | BF%, WC |
|  |  | AT | 0/16 |  | 3d/wk; 80%maximal aerobic speed; 50-60min; moderate intensity interval training |  |
|  |  | CON | 0/14 |  | regular PE/PA |  |
| Winwood et al.,2019 | 7 | ST | 39/0 | 14~15 | 2d/wk; : bodyweight, mobility training, and free-weights training. | CMJ |
|  |  | CON | 23/0 |  | regular PE/PA |  |
| Harris et al.,2021 | 8 | HITT | 41/12 | 10~13 | 2d/wk; 90% HR_max_; 15min; HIIT embedded in PE | BMI, BF%, push-ups |
|  |  | CON | 23/7 |  | regular PE/PA |  |
| Juric et al.,2023 | 12 | HIIT | 53/52 | 10~15 | 2d/wk; maximal exertion; 10min; jumping, burpees, and running | SLJ, 20-m sprint |
|  |  | CON | 53/49 |  | regular PE/PA |  |
| Marta et al.,2019 | 8 | ST | 19/0 | 10~11 | 2d/wk; 1- and 3-kg medicine ball throws, jumps onto a box (from 0.3-to-0.5-m-tall), and sets of 30-to-40-m-speed running. 45min; | SLJ, CMJ, 20-m sprint |
|  |  | CON | 18/0 |  | regular PE/PA |  |
| Robinson et al.,2022 | 4 | ST | 12/17 | 15.78 ± 0.44 | 3d/wk;4min; Tabata format | push-ups |
|  |  | CON | 15/8 |  | regular PE/PA |  |
| Song et al.,2012 | 12 | AT | 12/0 | 12~13 | 3d/wk; 60-70%HR_max_; 50min; walking, jogging, jumping, skipping, hoping, pumping, knee kicking, and sprinting | BMI, BF%, WC, VO_2max_ |
|  |  | CON | 10/0 |  | regular PE/PA |  |
| Cataldi et al.,2021 | 8 | HIIT | 9/6 | 18.26 ± 0.52 | 2d/wk; high intensity; 60min; whole body CrossFit program | BMI, WC, push-ups, 20-m sprint |
|  |  | CON | 9/6 |  | regular PE/PA |  |
| Velez et al.,2010 | 12 | ST | 8/7 | 16.1 ± 0.2 | 3d/wk; supervised strength training; 10RM; 35-40min; bench press, hammer strength seated row, shoulder press, and hammer strength squats | BMI, BF% |
|  |  | CON | 8/5 |  | regular PE/PA |  |
| Wong et al.,2008 | 12 | AT+ST | 12/0 | 13~14 | 2d/wk; 65% to 85% HR_max_; 45-60min; bodyweight, medicine balls, small side ball games, and stair-climbing ascending and descending exercises | BMI, BF% |
|  |  | CON | 12/0 |  | regular PE/PA |  |

**Table S4-S12:** Inconsistency result table in this network meta-analysis.

**Table S4:** Inconsistency result table for BMI.

Global inconsistency test: χ^2^ =4.33, *p* =0.931

Local inconsistency test:

**
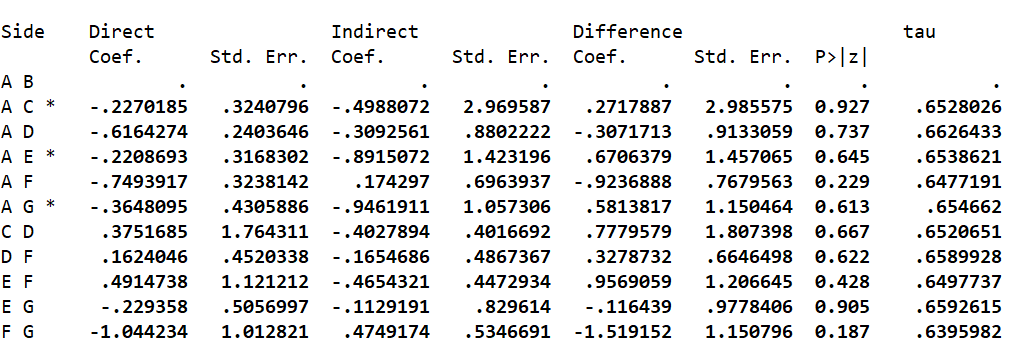
**

**Table S5:** Inconsistency result table for body fat percent.

Global inconsistency test: χ^2^ =6.16, *p* =0.802

Local inconsistency test:

**
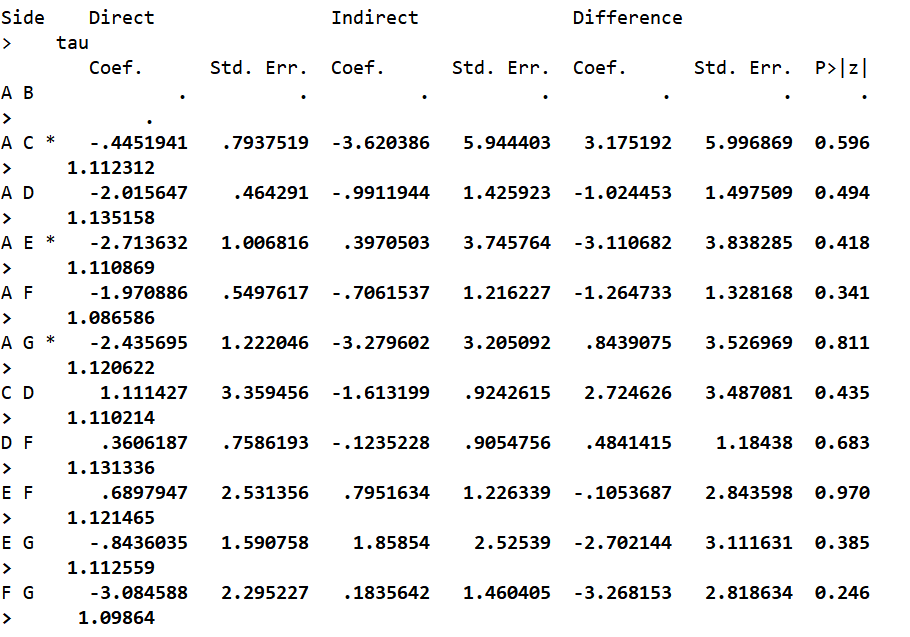
**

**Table S6:** Inconsistency result table for waist circumstance.

Global inconsistency test: χ^2^ =3.68, *p* =0.298

Local inconsistency test:

**
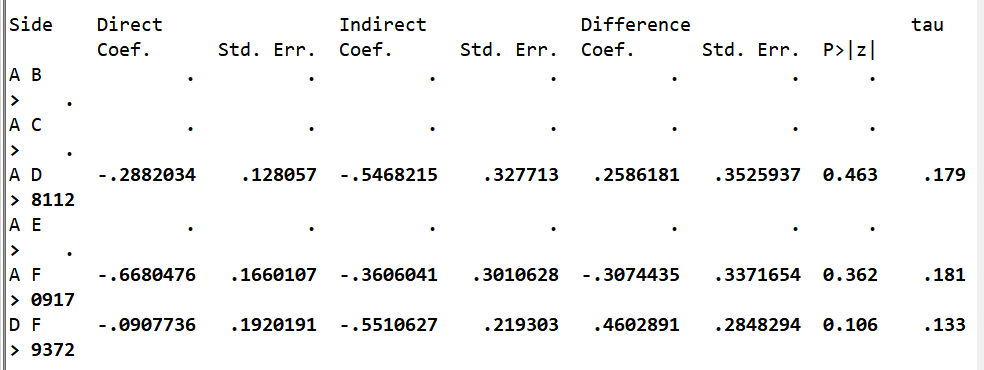
**

**Table S7:** Inconsistency result table for standing long jump.

Global inconsistency test: χ^2^ =1.64, *p* =0.977

Local inconsistency test:

**
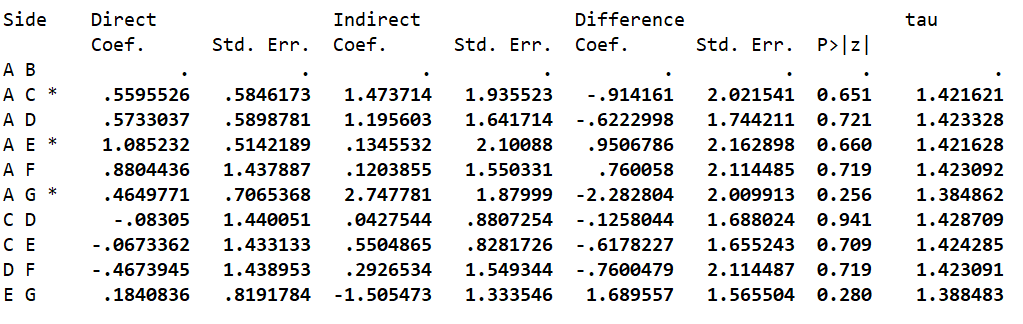
**

**Table S8:** Inconsistency result table for countermovement jump,

Global inconsistency test: χ^2^ =3.20, *p* =0.362

Local inconsistency test:

**
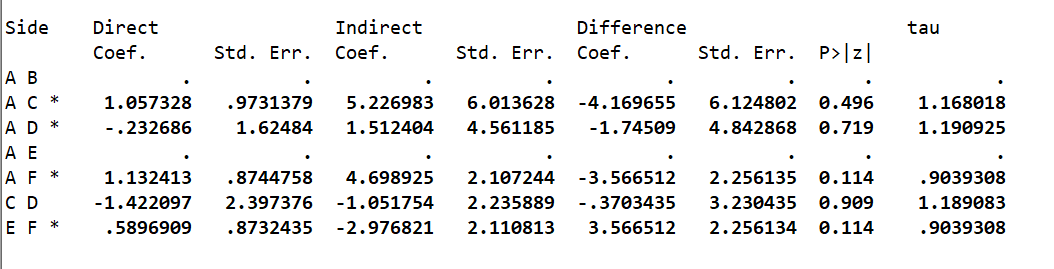
**

**Table S9:** Inconsistency result table for push-ups.

Global inconsistency test: χ^2^ =0.05, *p* =0.815

Local inconsistency test:

**
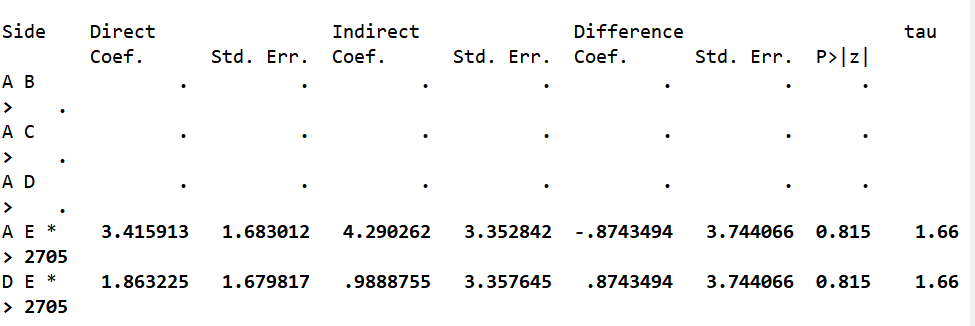
**

**Table S10:** Inconsistency result table for 20-m sprint

Global inconsistency test: χ^2^ =5.79, *p* =0.215

Local inconsistency test:

**
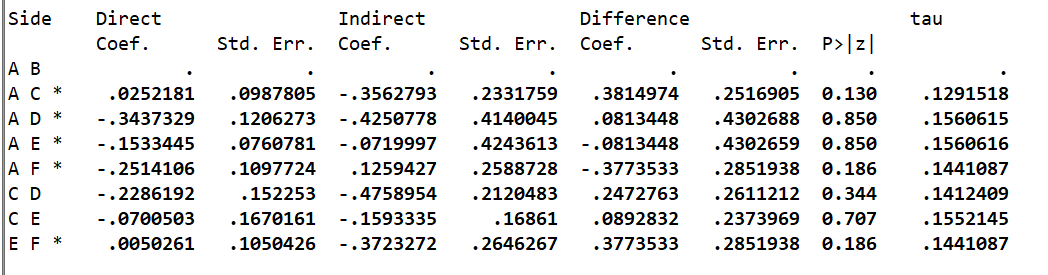
**

**Table S11:** Inconsistency result table for shuttle running.

Global inconsistency test: χ^2^ =1.83, *p* =0.767

Local inconsistency test:


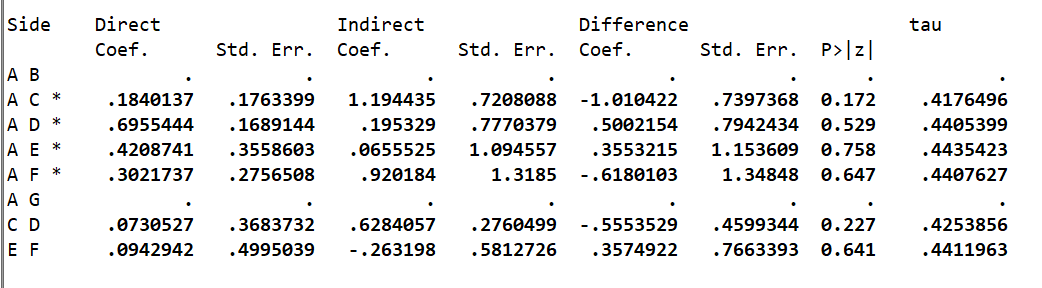


**Table S12:** Inconsistency result table for VO_2max_.

Global inconsistency test: χ^2^ =3.07, *p* =0.547

Local inconsistency test:

**
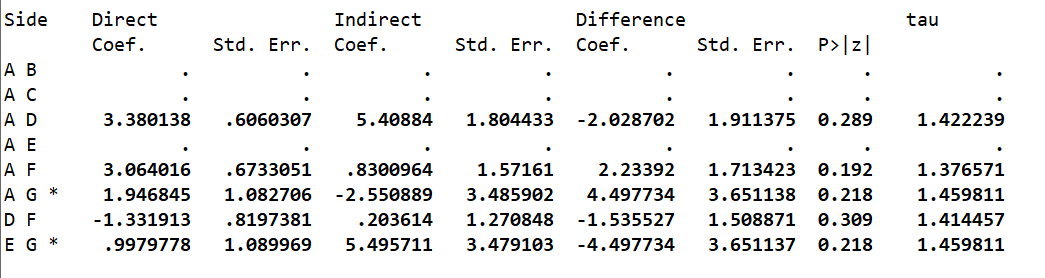
**
